# The Interaction of Fluorinated Glycomimetics with DC-SIGN: Multiple Binding Modes Disentangled by the Combination of NMR Methods and MD Simulations

**DOI:** 10.3390/ph13080179

**Published:** 2020-08-04

**Authors:** J. Daniel Martínez, Angela S. Infantino, Pablo Valverde, Tammo Diercks, Sandra Delgado, Niels-Christian Reichardt, Ana Ardá, Francisco Javier Cañada, Stefan Oscarson, Jesús Jiménez-Barbero

**Affiliations:** 1 CIC bioGUNE, Basque Research Technology Alliance, BRTA, Bizkaia Technology park, Building 800, 48160 Derio, Spain; jmartinez@cicbiogune.es (J.D.M.); pvalverde@cicbiogune.es (P.V.); tdiercks@cicbiogune.es (T.D.); sdelgado@cicbiogune.es (S.D.); aarda@cicbiogune.es (A.A.); 2 Centre for Synthesis and Chemical Biology, University College Dublin, Belfield, Dublin 4, Ireland; anginfantino@outlook.com (A.S.I.); stefan.oscarson@ucd.ie (S.O.); 3 CIC biomaGUNE, Basque Research Technology Alliance, BRTA, Miramon Park, 20014 San Sebastian, Spain; nreichardt@cicbiomagune.es; 4 Centro de Investigaciones Biológicas Margarita Salas, CSIC, Ramiro de Maeztu 9, 28040 Madrid, Spain; jcanada@cib.csic.es; 5 CIBER de Enfermedades Respiratorias (CIBERES), Avda Monforte de Lemos 3-5, 28029 Madrid, Spain; 6 Ikerbasque, Basque Foundation for Science, 48009 Bilbao, Spain; 7 Deptartment Organic Chemistry II, Faculty of Science and Technology, UPV-EHU, 48940 Leioa, Spain

**Keywords:** NMR, molecular recognition, glycans, lectin, STD-NMR, DC-SIGN, glycomimetics, CORCEMA, MD simulations, fluorinated carbohydrates

## Abstract

Fluorinated glycomimetics are frequently employed to study and eventually modulate protein–glycan interactions. However, complex glycans and their glycomimetics may display multiple binding epitopes that enormously complicate the access to a complete picture of the protein–ligand complexes. We herein present a new methodology based on the synergic combination of experimental ^19^F-based saturation transfer difference (STD) NMR data with computational protocols, applied to analyze the interaction between DC-SIGN, a key lectin involved in inflammation and infection events with the trifluorinated glycomimetic of the trimannoside core, ubiquitous in human glycoproteins. A novel 2D-STD-TOCSYreF NMR experiment was employed to obtain the experimental STD NMR intensities, while the Complete Relaxation Matrix Analysis (CORCEMA-ST) was used to predict that expected for an ensemble of geometries extracted from extensive MD simulations. Then, an in-house built computer program was devised to find the ensemble of structures that provide the best fit between the theoretical and the observed STD data. Remarkably, the experimental STD profiles obtained for the ligand/DC-SIGN complex could not be satisfactorily explained by a single binding mode, but rather with a combination of different modes coexisting in solution. Therefore, the method provides a precise view of those ligand–receptor complexes present in solution.

## 1. Introduction

Molecular recognition processes are essential for communication of biological entities. In this context, the three-dimensional structures of the partners that are involved in the interaction are strongly related to the corresponding biological response. Within biomolecules in nature, the chemical diversity of carbohydrates (oligosaccharides, glycans) endows them with a strong capacity for coding messages and translating them [1,2,3,4,5].

The last years have enjoyed large advances in the Glycoscience field [6,7,8,9,10,11], providing new tools and perspectives to expand the understanding on the structure-function relationship of the events mediated by sugar–lectin interactions. These developments have also led to important progress in the use of glycomimetics [12,13], sugar analogues devised to bind lectins, antibodies, or enzymes [14,15,16]. These synthetic molecules are structurally related to sugars although granted with altered physical chemical properties through structural modifications [17]. One of the most common alterations uses fluorine [18], the less abundant halogen in metabolites in nature, but highly present in drugs and pharmaceuticals [19,20], where it may boost adsorption and distribution properties [21].

In the NMR context, 19F spectroscopy is especially suitable for monitoring molecular recognition events. Besides, the intrinsic sensitivity of the 19F nucleus, 19F-NMR parameters provide additional chemical and structural information complementary to that achieved by using standard 1H and 13C- NMR [22]. Particularly in glycans, the tremendous overlap of 1H NMR signals can be overcome by introducing specific fluorine atoms in the molecule. Thus, glycomimetics decorated with fluorine atoms may be used in medicinal and pharmaceutical chemistry as well as in chemical biology and their interactions can be monitored by 19F-NMR experiments [23,24,25,26].

Among glycan binding proteins, C-type lectins mediate efficient immune responses. They feature broad recognition profiles that nevertheless can be precisely tuned with the chemical complexity of the saccharide. The Dendritic Cell-Specific ICAM-3-Grabbing Non-integrin, DC-SIGN, is one of the most widely studied C-type lectins. This lectin is highly abundant in dermal dendritic cells (DCs) [27,28,29], and is integrated in the asialoglycoprotein receptor-(ASGPR)-related and endocytic DC membrane receptors [30,31], which play key roles in innate immunity [29,32]. It is directly involved in HIV infectivity [33,34,35,36] and it is also crucial for the development of other pathogen-involved diseases, such as Ebola [37] and dengue [38,39,40], which has boost its attraction as a therapeutic target.

With respect to its glycan binding selectivity, DC-SIGN represents the paradigm of binding promiscuity [41,42,43]. This fact is strongly related to its 3D architecture. The primary Ca^2+^ site is located at a flat surface, exposed to the solvent and displays very few projecting amino acid sidechains. Thus, this construction enables the adaptation of a large variety of sugars. DC-SIGN preferentially binds D- mannose (Man) and L-fucose (Fuc), but also D-glucose (Glc), N-acetyl-D-glucosamine (GlcNAc), and N-acetyl-D-mannosamine (ManNAc) [42]. Despite this fact, its biological and biomedical interest have encouraged the design, synthesis, and applications of diverse glycomimetics.

**Figure 1:**
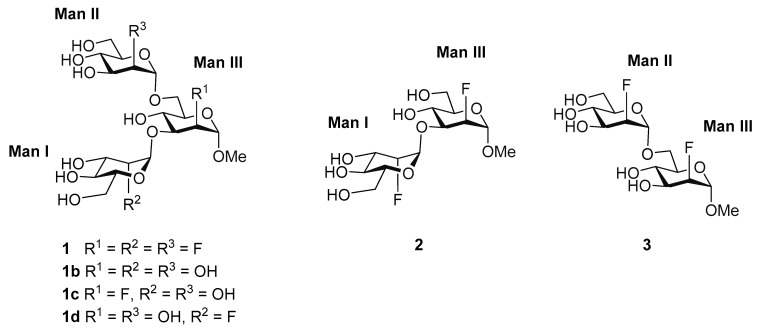
The trifluorinated trisaccharide glycomimetic **1** and its disaccharide components (**2,3**). The natural trimannoside core (**1b**) and related monofluorine-containing glycomimetics (**1c,1d**) are also shown. The non-reducing terminal residues are labelled as Man I (1→3 linkage) and Man II (1→6 linkage). The reducing (branched) residue in the trisaccharide is Man III.

The possibility of employing fluorinated glycomimetics to address their recognition features in comparison to those of their natural counterparts was explored in this study. In particular, we explored the binding features of a trifluorinated glycomimetic (**1**) of the ubiquitous trimannoside present in the inner core of N-glycans (**1b**), and its two constituent difluoro mannoside disaccharides **2** and **3** (Figure 1), to DC-SIGN by using an integrated approach of ^19^F-based STD-NMR spectroscopy methods and computational techniques. The novel 2D-STD-TOCSYreF NMR experiment [44] was employed, in combination with Complete Relaxation and Conformational Exchange Matrix Analysis of Saturation Transfer (CORCEMA-ST) and extensive Molecular Dynamics (MD) simulations, to deduce the binding epitope of each ligand. For that purpose, we designed an in-house computer program that is able to find the ensemble of structures that provide the best fit to the experimental NMR data. Remarkably, none of the experimental STD profiles obtained for each ligand bound to DC-SIGN can be satisfactorily explained by a single binding mode, but rather with a combination of different modes coexisting in solution.

## 2. Results and Discussion

The interaction of DC-SIGN with Man-containing oligosaccharides has been widely studied at the molecular level [43,45,46,47]. Man moieties interact with DC-SIGN by anchoring the Ca^2+^ at the primary binding site through O3-O4 (or its alternative O4-O3 pose) [45] or O2-O3 diols [48]. For high-Man ligands with the core trimannoside (**1b**) motif, the Manα1-3Man branch coordinates the Ca^2+^ ion of the lectin, whereas the Manα1-6Man branch is typically accommodated in a secondary site facing Phe313 (PDB codes: 1SL4, 1K9I) [43,47]. A different binding mode to DC-SIGN has been described (PDB: 2XR5) [49] for a pseudo-1,2-mannobioside ligand consisting of a Man moiety attached to a cyclohexane ring. In this case, the Man unit binds to the Ca^2+^ ion through the O3-O4 diol, while the cyclohexane ring folds towards the Val351 side chain, establishing van der Waals contacts.

In order to determine the bound conformations of each fluorinated glycomimetic to DC-SIGN, a protocol that combines the versatility of STD-NMR experiments with extensive computational data was adopted. First, 2D-STD-TOCSYreF NMR spectra [44] were recorded to take advantage of the increased resolution of these ^19^F-NMR experiments over the regular ^1^H-NMR based methods. In this manner, the experimental STD data, which contain the binding epitope information, were measured. In a parallel manner, the possible binding poses for each ligand, based on previously reported X-ray [43,45,47,49] and NMR data [46,48,50], were employed as starting geometries for extensive MD simulations. For every proposed binding mode, a large number of frames from the computed MD trajectories were submitted to CORCEMA-ST calculations [51,52] to predict their expected STD profiles. Finally, a computer program was developed to iteratively search for the best combination of MD frames that are able to quantitatively fit the experimental STD NMR data. As a result, the contribution of every binding mode present in solution was assessed.

### 2.1. 2D-STD-TOCSYreF Experiments and Affinity Measurements

**Figure 2:**
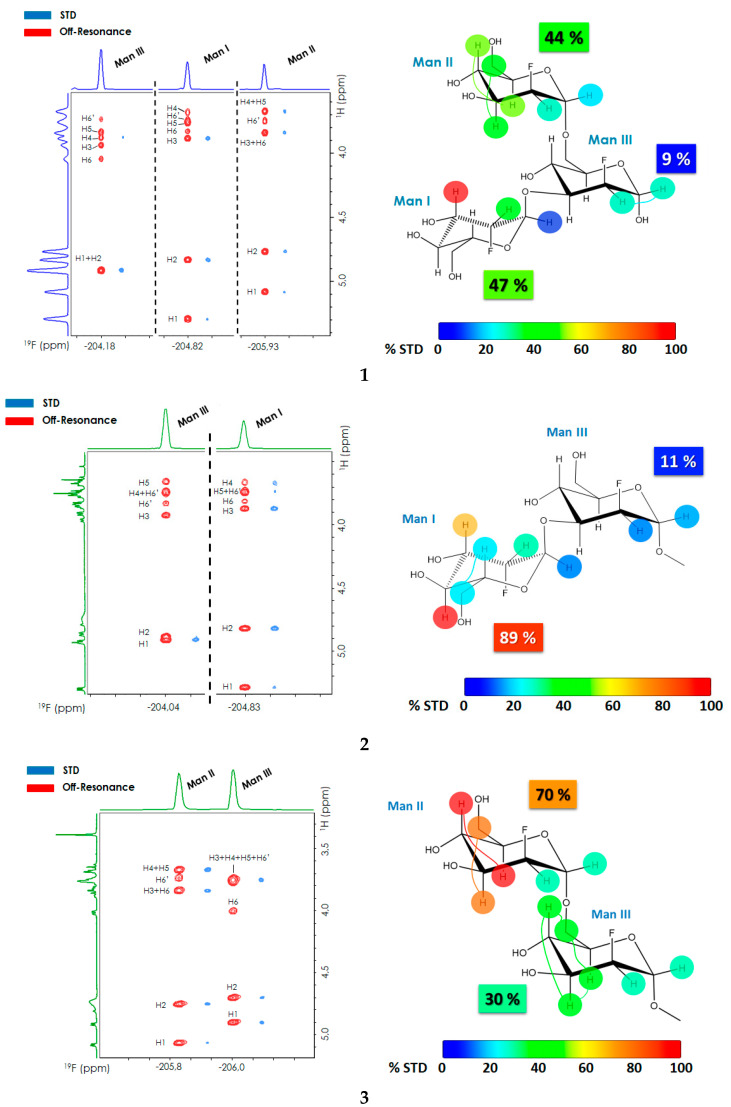
2D-TOCSYreF off-resonance spectrum (red) and 2D-STD-TOCSYreF peaks (blue) obtained for **1** (top panel), **2** (central panel) and **3** (bottom panel) in the presence of DC-SIGN. The ^19^F NMR frequencies are given in the abscissa axis and the ^1^H NMR frequencies in the ordinate axis. The STD cross peaks are shifted to high field in the ^19^F frequency to facilitate visual inspection. Every blue cross peak corresponds to the STD NMR intensity of the particular ^1^H NMR nucleus in a given monosaccharide, which is encoded by the ^19^F frequency. The ligand concentration was 1.4 mM in each case, with 9.14 μM of DC-SIGN tetramer, thus containing four carbohydrate recognition domains (CRD) (ligand/protein-CRD ratio is 38:1). The on-resonance irradiation was set to 0.85 ppm, with a saturation time of 2 s. The relative STD values, shown by colored circles, are obtained from the ratio between the corresponding peaks in the 2D STD-TOCSY-reF and 2D-TOCSYreF off-resonance spectra, and normalized to the largest STD in each molecule. The circles connected by a colored thin line correspond to overlapped signals in the spectrum, so that the STD intensity is shown as ‘spread’ between the corresponding ^1^H nuclei (meaning each of these protons may contribute or not to the total STD observed). In this case, the added STD intensity is given. The total relative STD per Man ring for each molecule is also specified with a colored tag, using the same color code as the circles.

The interaction between DC-SIGN and the three Man-based 2-deoxy-fluoro oligosaccharides (Figure 1) was first analyzed using 2D-STD-TOCSYreF experiments [44] (Figure 2). A long TOCSY mixing time (128 ms) was used to extend the polarization to all protons within the same spin system for each Man ring. The enhanced resolution of the 2D-TOCSYreF allowed the assignment of most of the STD NMR cross peaks with high reliability for the three fluorinated compounds (Figure 2 and Table 1). Generally speaking, the inherent loss of sensitivity due to the heteronuclear reF transfer in the 2D-STD-TOCSYreF experiments [44] is overcompensated by the gained spectral dispersion. Nevertheless, overlapping of some signals still occurred in **3**, as indicated in Figure 2.

The 2D-STD-TOCSYreF analysis of each molecular system revealed the parts of the ligand receiving efficient saturation from the protein (Table 1). For **1**, the terminal 1-3 and 1-6 branches exhibited the highest relative STD intensities (47% and 44%, respectively, normalized to the global observed STD), while the STD effects for the central Man III were notoriously weaker (9%). For disaccharide **2**, a large STD was observed at Man I (89% relative STD) versus Man III (11%), whereas for **3,** the observed STDs were 70% at Man II and 30% at Man III. Fittingly, these results agree to some extent with those observed for the trisaccharide **1**, whose STD profile is roughly the addition of the observed STDs for the two disaccharides. Noteworthy, the STD displayed by the central Man III is much lower than that of the terminal Man I and Man II units. Indeed, this behavior is also noticeable on the dimannosides **2** and **3**, for which the reducing Man III unit always displays the lowest STD effects. In all cases, H3 or H4 of either Man I (in **1** and **2**) or Man II (in **3**) always showed the highest STD NMR intensity when on-resonance saturation was set at the aliphatic region (0.85 ppm).

Although the experimental information obtained is reliable, it is not straightforward to translate it into a particular binding pose. As mentioned above, multiple binding modes have been widely observed for Man-containing ligands when interacting with DC-SIGN. In particular, Man moieties may interact with DC-SIGN through direct coordination of the Ca^2+^ via O3-O4 or the ‘inverse’ O4-O3 [45], and also through the O2-O3 diol [48]. Although the presence of a fluorine atom at position C-2 in **1**-**3** precludes this binding mode herein, the possible binding poses for these ligands in solution are still numerous.

In addition to the binding epitope, the binding affinities of **1-3** to DC-SIGN were also deduced by competitive ^19^F-R_2_ filtered NMR experiments, using 6-F-ManαOMe (K_D_ 1.3 mM) as a spy molecule (see experimental section and Figure S1 in SI). In order to gain insights into the effects that the different fluorination patterns may have in the binding event, the affinities of **1b**, the natural analogue of **1**, as well as **1c** and **1d**, two partially fluorinated trisaccharides, were also estimated. (Table 2, Figure S1).

The results indicate that the best binders are the natural molecule **1b** and the two monofluorinated analogues, **1c** and **1d**, which essentially display the same affinity. The trifluorinated trisaccharide **1** is two-fold weaker, while the difluorinated disaccharides **2** and **3** are nine- and four- fold weaker ligands, respectively.

Interestingly, mono-fluorination at C-2 of **1b** at the branched Man III (to give **1c**) or at the terminal Man I (to give **1d**) do not appreciably influence affinity. However, when all the three Man moieties are fluorinated (**1**), the affinity drops by half. In a qualitative manner, these data suggest that, individually, the contribution of Man I and Man III’s OH-2 to the interaction with the lectin is negligible, while that of Man II is relevant. Whether the loss in affinity observed for **1** is caused by the fluorination of Man II or rather by a combined effect of the simultaneous fluorination of the three Man rings, cannot be directly inferred solely from these affinity measurements.

### 2.2. Molecular Dynamics Simulations

Molecular Dynamics simulations were then employed to provide possible 3D models of the complexes between DC-SIGN and each fluorinated glycomimetic. As mentioned above, X-Ray crystallography and NMR studies have demonstrated that Man-containing saccharides may bind this lectin in different poses, given the diverse possibilities of the Man O3 and O4 atoms to coordinate the key Ca^2+^. Therefore, several starting geometries were generated for the complexes of glycomimetics **1**-**3** with DC-SIGN, by considering the following restraints:

- The vicinal O3 and O4 atoms of any Man residue coordinate the key Ca^2+^.
- The corresponding Man moiety was superimposed with the equivalent Man unit deposited on PDB 2IT5 [45]. This crystallographic structure contains two alternative O3-O4 (major) and O4-O3 (minor) poses, respectively.

Thus, there are four potential binding modes for **1** (O3-O4 and O4-O3 alternatives at Man I and the equivalent ones at Man II). Similarly, there are four potential poses for **3** (through Man II and Man III) while there are only two (through Man I) for analogue **2**, since Man III O3 is now involved in the glycosidic linkage. Overall, 10 different starting geometries of the DC-SIGN-glycomimetics complexes were tested and analyzed by long MD simulations in explicit water.

**Figure 3:**
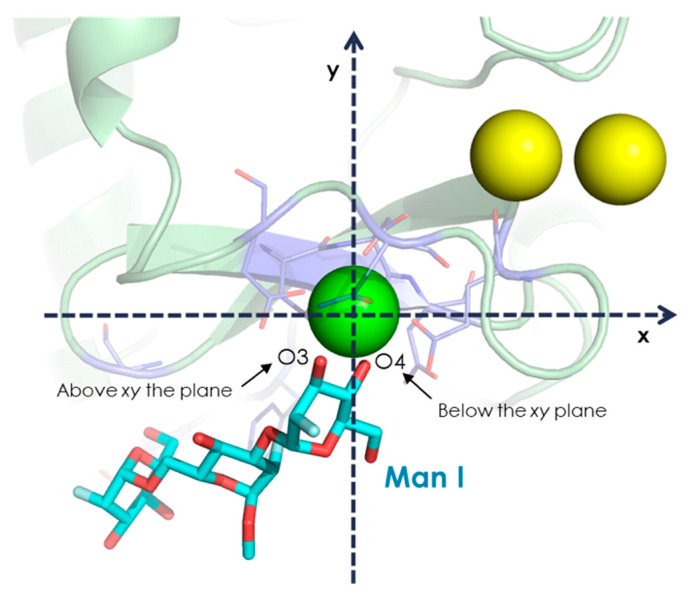
Schematic view of one possible complex between **1** and DC-SIGN describing the employed Cartesian system to describe the coordinating oxygen atoms. The geometric center of the three Ca^2+^ ions of DC-SIGN is on the *xy* plane, the key Ca^2+^ (green sphere) is set at the origin, and the other two ions (yellow spheres) are placed in the first quadrant. The coordinating oxygen closer to the viewer is first specified (O3 in this case), and the other one (O4) is named later. Therefore, this binding pose is “ManI_O3-O4”.

In order to classify the different binding poses, a nomenclature for each ligand was defined as follows: “name of the Man unit interacting with Ca^2+^ + identifier of the coordinating oxygen closer to the viewer – identifier of the other coordinating oxygen“ (Figure 3). For instance, the two possible binding modes for **2** are “ManI_O3-O4” and “ManI_O4-O3”. The modelled complexes are gathered in Table 3, while their corresponding 3-D structures are given in Figure S3.

The stability of the different complexes during the MD simulations was evaluated. In general, the major stabilizing contacts with the lectin involve the Man unit that coordinates the Ca^2+^ ion either via O3-O4 or O4-O3. A well conserved hydrogen bond (HB) network is observed for the three glycomimetics in all binding poses. The OH groups at positions 3 and 4 establish HB contacts with Glu354, Glu347, Asn365 (>80% of simulated time in which the complex is associated), and Asn349 (50–80%). More specifically, for the O3-O4 binding modes, OH-3 contacts the side chains of Glu347 and Asn349, whereas OH-4 interacts with Glu354 and Asn365. These interactions are swapped for the O4-O3 poses (see Figure S5 in the SI for a detailed summary of all interactions).

The most stable binding mode found for **1** was ManI_O3-O4, where the ligand adopts an extended conformation which resembles those present in PDB structures 1SL4 and 1K9I [43,47]. In the alternative O4-O3 pose, the ligand is folded over Val351, as found for the pseudo-1,2-mannobioside in 2XR5. Several van der Waals contacts are detected along the simulations, mainly involving H-3, H-4, and H-5 of any of the three Man moieties, depending on the particular binding mode (Figure S5). Finally, for the ManII_O3-O4 pose, the ligand is mostly solvent exposed, since the large mobility of the 1-6 branch hampers the existence of stable interactions of the rest of the ligand with the lectin side chains.

Regarding the fluorinated dimannoside **2**, the binding mode ManI_O3-O4 is the most stable and somehow similar to the equivalent in **1**, but since it lacks the Man II unit, the ligand displays more mobility over the lectin surface. In the alternative pose, the less stable ManI_O4-O3 geometry, occasional van der Waals contacts of Man II H-4 and Man I H3 with Val351 are detected (10% of simulated time), and the ligand is more solvent exposed (Figure S5).

Finally, **3** shows a rather variable stability for its different binding modes. Remarkably, the ManII_O3-O4 geometry binds very tightly in the simulations (400 ns of association time on average) and displays HBs involving OH-3 and OH-4 of Man II and Glu358. A CH-π between Phe313 and H-3 Man III is also present for most of the simulated time (Figure S5).

### 2.3. CORCEMA-ST and Best-Model STD Fitting

As mentioned above, the interpretation of the entire set of experimental STD NMR intensities in terms of ligand/lectin molecular complexes is not straightforward, even considering the MD simulations described above. Thus, a quantitative approach was applied. First, the computed MD trajectories were submitted to a COmplete Relaxation and Conformational Exchange Matrix Analysis of Saturation Transfer (CORCEMA-ST) analysis [51,52] to estimate the expected STD-NMR intensities. Interestingly, CORCEMA-ST has been previously employed to explore multiple binding events in sugar-lectin complexes. As leading examples, the dual binding mode of Man-containing saccharides to DC-SIGN [46,50] has been shown, as well as different interaction possibilities of Tn-bearing glycopeptides to bind MGL [53].

Then, an in-house computer program dubbed *BM-Mixer* was developed and applied to fit the theoretical to the experimental STD NMR values. This protocol, which might be of general application, allowed the contribution of the different binding modes of each ligand to be dissected when bound to DC-SIGN. In particular, the procedure employs the complete MD trajectory of each modelled binding mode to fit the STD data in a fully automated manner (see experimental section for a detailed view of the protocol).

**Figure 4:**
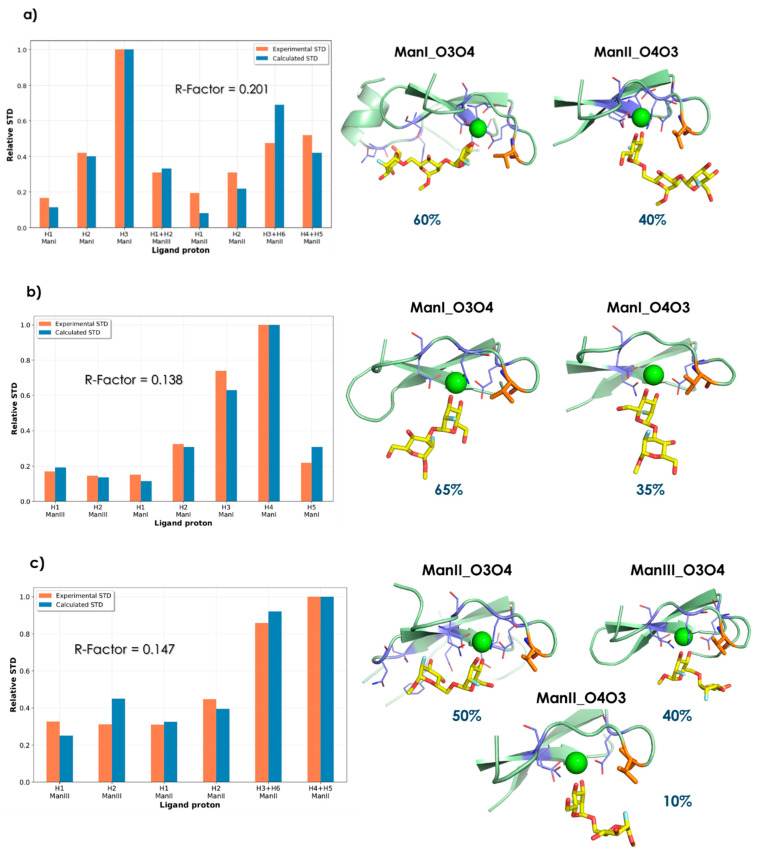
Contributions of the individual binding modes that provide the best fit to the experimental STD NMR data. Representative structures along with the associated NOE R-Factor_rel_ of the best binding mode combination found for compounds **1a**, **2b,** and **3c** are shown. Val351, which is the only aliphatic residue at the lectin binding site, is colored in orange.

*BM-Mixer* calculations were performed using ensembles of 400–800 frames per simulated binding mode. Only reliable experimental STDs were used in the calculation, to avoid the introduction of noise. In cases of isochronous NMR frequencies, the observed experimental STD was considered as the sum of the individual contributions from each signal (see Section 3.5 in “Material and Methods” part). The fractions of each binding mode of **1**-**3** to DC-SIGN that better fitted the experimental STD data are shown in Figure 4 (see also Table S1). Globally speaking, the predicted contributions for each binding mode agree with the relative stability observed in the MD simulations of each ligand (Table 3).

According to the computational procedure for the complex of trimannoside **1** with the lectin, the best fit was obtained with a combination of 60% ManI_O3-O4, and 40% ManII_O4-O3 (Figure 4a). Thus, both Man I (major) and Man II (minor) directly interact with the calcium at the binding site. For the ManII_O4-O3 geometry, stabilizing intermolecular van der Waals contacts are predicted with Val351. Qualitatively, the presence of this geometry in the distribution allows an explanation for the strong STD observed for Man I H-3. In contrast, the observed STDs at Man III (H-1/H-2) are probably accounted for by its vicinity to Phe313 in the major ManI_O3-O4 geometry. Remarkably, the most populated binding modes found by *BM-Mixer* are similar to those of the deposited X-ray structures 1K9I and 1SL4 (ManI_O3-O4, 60%) and 2XR5 (ManII_O4-O3, 40%). Fittingly, *BM-Mixer* is able to find the best solution taking from the complete MD ensemble calculated for each binding mode instead of using just a limited number of representative structures.

**Figure 5:**
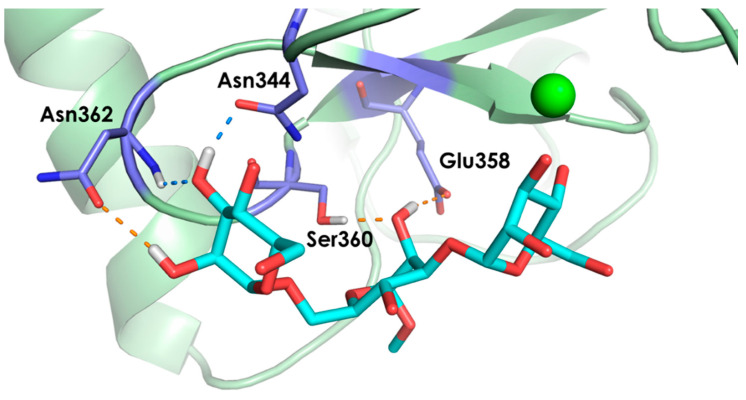
Possible intermolecular hydrogen bond interactions predicted by the MD simulations for the complex between DC-SIGN and the core trimannoside **1b.** These hydrogen bonds (HBs) are absent in the corresponding complex with the trifluorinated glycomimetic **1.** Hydrogen bonds involving OH-2 groups of Man II and Man III are colored orange, and the ones involving OH-3 are in blue. The most stable binding pose, ManI_O3-O4, was used as starting geometry.

Interestingly, the predicted major pose, ManI_O3-O4, is the only one in which several potential interactions involving the substituent at C-2 with the lectin side chains could take place. Thus, the corresponding geometry was built for the natural analogue **1b** bound to the lectin, and submitted to MD simulations. The analysis of the trajectory revealed the existence of intermolecular HB interactions involving OH-2 groups, obviously absent in the trifluorinated glycomimetic **1**. For the major ManI_O3-O4 geometry, OH-2 of the terminal Man II acts as a HB donor to Asn362 during 40% of the simulation time. Furthermore, HB contacts between OH-3 of the same Man II with Asn344 and Asn362 are more populated in **1b** than in **1** (70% versus 10%). Additionally, the MD simulations carried out for **1b** predict that OH-2 of the central Man III may be involved in HB interactions with Ser360 (as a donor) and with Glu358 (as an acceptor) around 70% of the simulation time (Figure 5). In fact, the averaged complex stability of trifluorinated **1** in the MD simulations is significantly smaller (lower association time) than that of **1b** (>400 ns in 5 MD replicas). The mobility of the ligand at the binding site is also much higher for **1** than for **1b** (see Figure S6 for a detailed summary of the interactions and Figure S7 for a summary of the computed torsion angles and root-mean-square Deviation (RMSD) plots).

For the complex of DC-SIGN with **2**, with O3 of Man III involved in the 1-3 linkage, only two poses are possible. The best fit was found for a population distribution of 65% ManI_O3-O4 and 35% of the alternative ManI_O4-O3 pose. The obtained major geometry is in agreement with that described above for the trisaccharide **1**, while the intermolecular contacts are also analogous, with the obvious exception of those that involve Man II. Indeed, the experimental STD profile for Man I is analogous to that observed for **1**, with the added contribution of H-4 arising from the alternative ManI_O4-O3 pose.

In the complex of compound **3**, Man III can also participate in the coordination of the Ca^2+^. In fact, the best fit is provided by a large contribution of the ManII_O3-O4 (50%) and ManIII_O3-O4 (40%) poses, with a small participation of the ManII_O4-O3 geometry (10%). The latter was predicted to a larger extent for the complex of trisaccharide **1**. However, in that case, Man I provided contacts with Val351 further stabilizing the pose. Interestingly, the major ManII_O3-O4 geometry for **3** cannot take place in **1**, given the presence of Man I, which would collapse with the protein surface.

Moreover, the analysis of the geometries permits an explanation of the observed relative affinities. As described in Table 2, the trifluorinated ligand **1** displays about half the affinity of its natural counterpart **1b**, and its monofluorinated analogues **1c** and **1d** versus DC-SIGN. Therefore, it is likely that the predicted hydrogen bonds involving OH-2 and OH-3 at Man II are at the heart of the observed loss of binding energy. In any case, the combined effect caused by the simultaneous fluorination of the three Man units is rather low in terms of binding energy (2.5 fold variation in binding affinity amounts to ca. 1 kcal/mol in binding free energy). The lack of Man II in **2** and of Man I in **3** precludes a number of stabilizing HBs and van der Waals contacts that lead to the observed loss in affinities in the fluorinated disaccharides versus the trisaccharide **1** (ca. four-fold **1** versus **2** and ca. two-fold **1** versus **3**).

## 3. Materials and Methods

### 3.1. Man-Based Ligands

The synthesis of compounds **1**, **1b, 2**, and **3** has already been described [44]. The synthesis of **1c** and **1d** is detailed in SI and Scheme S1. ^1^H/^19^F-NMR assignments for **1, 2,** and **3** are compiled in Table S2, and where previously described in [44].

### 3.2. DC-SIGN ECD Preparation

Plasmids pET15b holding the DC-SIGN full extracellular domain (ECD, residues 70-404, Thermo Fischer Scientific) were amplified in E. coli DH5α and subsequently transformed on BL21/DE3 competent cells (Sigma-Aldrich). A single colony was added to a preinocule of 100 mL, in the presence of 100 mg/L ampicillin, and grown overnight at 37 °C under continuous shaking. Then, each preinocule was diluted up to 1 L of Luria-Bertani (LB) broth containing 100 mg/L ampicillin, and cell growth was maintained at 37 °C with gentle shaking until OD = 0.60–0.65. Cultures were finally induced with 1 mM isopropyl-1-thio-b-D-galactopyranoside (IPTG) and allowed to grow overnight at 20 °C. The resulting pellets were harvested by centrifugation (4 °C, 4500 rpm, 20 min) and resuspended in the minimal amount of Tris-HCl buffer 10 mM (pH = 8.0). Cells were lysed by sonication and the unfolded ECD was separated in the insoluble fraction by ultracentrifugation (4 °C, 30 k rpm, 60 min). The isolated inclusion bodies were further solubilized in 6 M urea and 2 mM β-mercaptoethanol (0.01% v/v). By ultracentrifugation (4 °C, 40 k rpm, 180 min), the remaining insoluble cell debris were sedimented and the supernatant containing the unfolded ECD was carefully decanted. Protein refolding was performed by stepwise dialyzing the supernatant against 2 L of 100 mM Tris-HCl buffer (NaCl 150 mM, CaCl_2_ 10 mM, pH = 8.0, 24 h each step), containing 4 M urea, 2 M urea, and no urea, respectively. The ECD was first purified by affinity chromatography in a Mannose–Sheparose column, using as loading buffer Tris-HCl 20 mM, NaCl 150 mM, CaCl_2_ 10 mM, pH = 8.0; and as elution buffer, 20 mM Tris-HCl, 150 mM NaCl, 10 mM EDTA, pH = 8.0. Eluted fractions containing significant amounts of the ECD (A(λ_280_) > 0.10) were mixed and repurified by size exclusion chromatography (AKTA sys., GE Healthcare) in a HiLoad 26/600 Superdex 200 column (UV_max_ at V_elut_ = 140 mL, flow rate 2.5 mL/min) using as elution buffer Tris-HCl 20 mM, NaCl 150 mM, EDTA 1 mM, pH = 8.0. Conditions of the collected pure ECD fractions were changed to Tris-d_11_ 20 mM, CaCl_2_ 4 mM, NaCl 150 mM, pH = 8.0 (D_2_O), using Vivaspin membrane filters of 100k MWCO. The tetrameric state of the lectin was previously confirmed by TEM [48]. The presence of the protein was monitored throughout the entire protocol by 4–12% SDS-PAGE, and ECD concentrations were determined by UV-Vis spectroscopy (ε_280,tetramer_ = 280,600 M^−1^ cm^−1^, estimated from ProtParam).

### 3.3. NMR Experiments

All spectra were acquired at 298 K on either a Bruker AV500 or AV600 NMR spectrometers, equipped with a ^19^F probe (5 mm SEF ^19^F-^1^H with Z gradient). All the samples were prepared using the same protein buffer (20 mM Tris-d_11_, 4 mM CaCl_2_, 150 mM NaCl, pH = 8.0, in D_2_O) was employed. For molecular recognition experiments, controls were performed in the presence of either EDTA to sequester the Ca^2+^ or a known DC-SIGN ligand, to assess specific binding at the primary binding site.

Samples for the 2D STD-TOCSY-reF contained 9.14 µM ECD (tetramer) and 153 eq. of ligand (1.4 mM, the ratio ligand/protein (CRD) is 38:1) in each case. The 2D STD-TOCSY-reF setup consists on a proton saturation transfer scheme (^1^H_protein_→^1^H_ligand_-STD) with subsequent evolution through J_HH_ couplings (TOCSY), to finally refocus the STD-edited information in the ^19^F dimension (reF). As STD parameters, selective saturation was applied for 2 s using 90º PC9 pulses of 10 ms length, with on-resonance saturation set at 0.85 ppm and off-resonance at 60 ppm. The TOCSY mixing time used was 128 ms, and the corresponding J_HF_ evolution and refocusing delays in the reF module were Δ^H^ = 10.4 ms and Δ^F^ = 6 ms, respectively. The relative saturation values (%) were calculated from the ratio between peaks in the 2D STD-TOCSY-reF spectrum and the corresponding ones in the off-resonance TOCSY-reF spectrum. The values were normalized by assigning a 100% value to the peak that displayed the highest STD ratio.

^19^F-NMR R_2_/T_2_ experiments for affinity measurements were carried out employing a Carr-Purcell-Meiboom-Gill (CPMG) pulse sequence as follows: $\left[D-90_{x}-{\left(\tau -180_{y}-\tau \right)}_{n}-acquire\right]$, with a recovery delay D=4 s, and a list of 18 values for the spin-echo loop n to yield $T_{2}$ by fitting to $I\left(t\right)=I_{0}e^{-n2\tau /T_{2}}$ [48]. For K_D_ estimation of 6-deoxy-6-F-Man (the spy-molecule), the free evolution delay was set to an optimized value τ=1 ms, after using relaxation dispersion experiments to assure a negligible chemical exchange contribution to R_2,obs_ (Figure S1a). The K_I_ of **1**, **1b-d**, **2**, and **3** with DC-SIGN (ECD) was determined by titration experiments in a competitive fashion [54,55]. Increasing amounts of the competitor molecules (compounds **1**, **1b-d**, **2**, and **3**) were added to a solution mixture of the spy molecule and the protein in each case, calculating the R_2,obs_ of the spy molecule at each competitor concentration while the ratio [Spy-molecule]/[Protein] is fixed (40:1) (Figure S1c).

### 3.4. Molecular Dynamics Simulations

All compounds were initially built using Glycam-web tool [56]. The fluorinated glycomimetics **1**, **2,** and **3** were generated by modifying the Glycam structures with Discovery Studio or PyMol according to the particular molecule. Then, the fluorinated structures were submitted to energy minimization, charge and atom type assignment using Antechamber [57].

The deposited protein structure on PDB 2IT5 [30] was used as the lectin geometry for all MD simulations, after removing all but one CRD with its corresponding structural Ca^2+^ ions. The two models of the crystalized Man ligands coordinating the Ca^2+^ at the primary binding site served as templates for superimposing the starting geometries for the O3-O4 (most populated ligand orientation on 2IT5) and O4-O3 (less populated one) 3D models. Then, the crystalized ligands on the original 2IT5 structure were removed as well.

Overall, 10 models of DC-SIGN in complex with the ligands **1** (4 binding modes), **2** (2 binding modes), and **3** (4 binding modes) were built according to the criteria specified in the MD simulations section on *Results and Discussion* (also see SI). Additionally, a model of DC-SIGN with the natural analogue **1b** in the binding pose ManI_O3-O4 (see *Results and Discussion*) was also prepared.

All MD simulations were conducted using AMBER molecular dynamics simulations software (version 16). The employed preparation, minimization and production protocol was the same for all the simulations. First, solutes (either ligand or protein-ligand complexes) were solvated using a pre-equilibrated TIP3P rectangular water box, spanning 10 Å from the solute in each direction. Cl^-^ ions were added to neutralize the system, using Li/Merz parameters [58]. The AMBER’s ff14SB force field [59] was used to parametrize the protein, while either GAFF2 [60] (for analogues **1**, **1b–d**, **2,** and **3**) or GLYCAM06 [56] (for **1b**) parameters were employed for the ligands. The system was minimized in two stages, starting with the solvent and counterions while keeping the solute fixed, and following with a general minimization of the whole system. Then, gradual heating from 0 to 300 K in the NVT ensemble for 100 ps was performed. Further equilibration was run at 300 K in NPT during 1 ns, employing a Langevin thermostat with collision frequency 1 ps. The production dynamics were run in the NPT ensemble at 300 K, 1 bar, using the Langevin thermostat as in the previous stage. SHAKE was employed to constrain bond lengths on hydrogen atoms during production dynamics, and the time step was set to 2 ps. The cutoff for non-bonded interactions was set to 10 Å, and the Particle Mesh Ewald Method was used to introduce long-range electrostatic effects. The simulation time of the complexes in water was set to 400 ns, and 6 to 12 replicas were run in each case.

Additional MD simulations of 500 ns of the ligands **1** and **1b** in explicit water were also conducted, to assess the average conformational dispositions of the free molecules in solution (Table 2). The MD protocol employed was the same as beforementioned.

### 3.5. CORCEMA-ST and Best-Model STD Fitting

CORCEMA-ST allows prediction of the theoretical STD-NMR intensities of the ligand protons in a given receptor-ligand complex [51,61,62]. The STD profile depends on the geometry of the complex, rotational motion correlation times of the system, irradiation conditions, and dissociation constant, among others.

The following protocol was employed. For every ligand, a subset of 400–800 frames extracted from the MD trajectories computed for each binding mode (BM) was randomly selected. Only the BMs whose averaged association time in the MD simulation was larger than 5 ns were analyzed. The predicted STDs for each individual frame according to CORCEMA-ST were compiled and submitted to a Python based program, dubbed *BM-Mixer*, which aims to find the minimum ensemble of conformations of the bound ligand that best fits the experimental STD data, in an iterative manner. This fitting is guided by the minimization of the relative NOE R-factor (agreement factor), which is defined as follows for the ensemble of binding mode geometries [52]:

(1) $$NOE\;R-factor_{rel}=\sqrt{\frac{\sum_{i=1}^{k}{\left(STD_{exp,rel,i}-{\bar{STD}}_{teor,rel,i}\right)}^{2}}{\sum_{i=1}^{k}{\left(STD_{exp,rel,i}\right)}^{2}}}$$

where $STD_{exp,rel,i}$ is the experimental STD value of proton i normalized to the most intense experimental peak, and ${\bar{STD}}_{teor,rel,i}$ is the calculated averaged STD value of proton i, normalized to the most intense calculated peak for the particular combination of binding mode frames. The best-model STD fitting outcome converged for a minimum number of frames used in the calculations. In general, it was observed that a number between 400–800 frames was sufficient to yield convergence, depending on the ligand mobility at the binding site.

**Figure 6:**
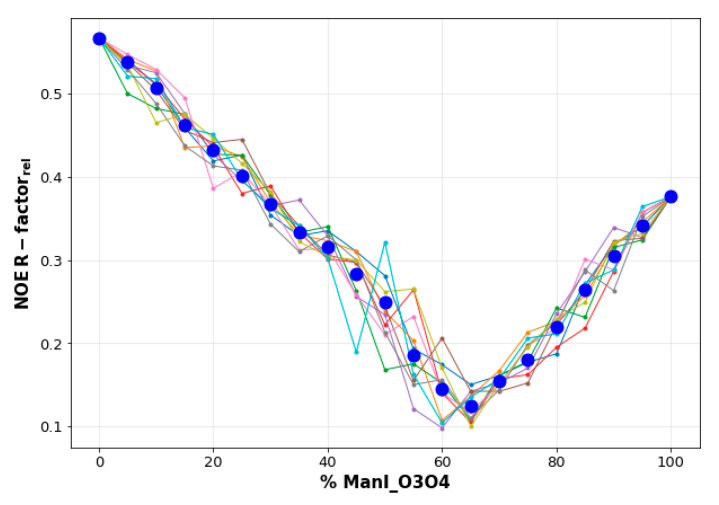
The fit of the NOE R-factor_rel_ value computed by *BM_Mixer* to the relative population of ManI_O3O4 frames. Obviously, the percentage of the alternative binding mode ManI_O4O3 is   100−%ManI_O3O4). The quantitative STD data predicted by CORCEMA-ST for 800 frames from the MD simulations were employed. Each colored line represents a different iteration of the main loop of the program (only 10 out of 20 are shown for clarity). Blue circles represent the mean value among all iterations for that particular combination of binding modes (BM). The minimum is reached for an ensemble of structures formed by 65% of ManI_O3O4 and 35% of ManI_O4O3. All the assigned experimental STD intensities were used in the calculations. In this case, 400 frames from the ManI_O4O3 trajectory and 400 frames from the ManI_O3O4 trajectory were employed.

The results of the search for the best combination of binding modes was now exemplified for **2.** Conveniently, only two binding modes are possible for this ligand. Figure 6 shows the evolution of the 20 iterations required to screen the space of possible BM combinations, expressed as the percentage of each BM in the *x*-axis. As it can be observed, the agreement between the calculated and the experimental STD data is rather poor when either of the two ensembles of structures representing each binding mode (100% ManI_O4O3 or 100% ManI_O3O4) are separately considered. However, the NOE R-factor_rel_ converges to a minimum when 65% of the ManI_O3O4 binding pose is considered.

The list of experimental STDs provided to *BM-Mixer* is crucial to obtain reliable results. It is essential to use a large enough number of experimental STD peaks to minimize possible ambiguities. It was observed that different BM combinations may provide a similar NOE R-factor_rel_. Since errors for very weak experimental STD signals (close to zero STD) may be rather high, it is advisable to discard these signals. Thus, the list of experimental STDs used for the calculations should only contain the reliable assigned STD peaks. Notably, the very weak peaks can be introduced in the computation a posteriori as internal controls for detecting false positives.

Obviously, it is convenient to examine whether each individual MD ensemble provides STD profiles that are different enough. Otherwise, *BM-Mixer* will provide an ambiguous fractional contribution of the possible binding poses. In these cases, additional experimental information may be required to differentiate between them.

For those peaks that are virtually isochronous in the NMR spectrum, the experimental STD intensity observed can be considered as the sum of the individual contributions of each individual proton (see Figure 5). *BM-Mixer* handles this ‘aggregated’ experimental STD file and automatically performs the corresponding sum of contributions of the CORCEMA-ST predicted STD values for the overlapped protons. In this manner, both calculated and experimental STD aggregate can be evaluated using the agreement factor. It was assumed that the shapes of the NMR signals are similar, and the protons are isochronous. Nevertheless, if the overlapped protons display rather different T_2_ relaxation times or their resonance frequencies are not exactly the same, considerable errors could be introduced in the calculations. For instance, in the case of compound **3**, the experimental STD cross peak observed for Man III at 3.7 ppm could correspond to H-3, H-4, H-5 or H-6′. Since considering the measured STD intensity as the sum of the individual contribution from four H atoms would introduce noise in the search, the corresponding STD peak was not considered in the best-model fitting shown in Figure 5.

Since CORCEMA-ST is designed to calculate the absolute intensity of 1D ^1^H-STD-NMR spectra, some considerations were taken to assess its application to the analysis of the 2D-STD-TOCSYreF experiments. In particular, the experiment employed herein includes a homonuclear ^1^H-TOCSY block and a heteronuclear ^1^H,^19^F reF transfer. Thus, the absolute STD intensities are not directly comparable to those of the regular 1D-STD-NMR experiment. However, the relative STD profiles (i.e, the STD intensities normalized to the most intense STD peak) are expected to remain similar. Regarding the employment of the ^19^F nucleus, since the ^1^H→^19^F heteronuclear cross-relaxation is largely less efficient than the ^1^H→^1^H homonuclear one, it is not expected that its effect is noticeable. In any case, as control, CORCEMA-ST calculations were also performed using molecular models the ^19^F atoms were replaced by ^1^H. Fittingly, these CORCEMA-ST controls demonstrated that the predicted contribution of each binding mode that provided the best NOE-R-factor_rel_ was rather insensitive to the presence of the F atom (Table S3).

## 4. Conclusions

A new protocol for unraveling multiple binding modes is presented, applied for fluorine containing molecules but which might be used for all types of molecules. The protocol is based on the synergic combination of the novel 2D STD-TOCSY-reF experiment to extract reliable STD values with extensive MD simulations. Finally, a computer program, *BM-Mixer,* was able to find the combination of geometries from the complete MD ensemble of the ligand/protein complex that provided the best match to the experimental data. The interaction of the trifluorinated mimetic of the trimannoside core present in mammalian glycoproteins with DC SIGN a key lectin involved in infection diseases was studied. The results indicate that no single solution can account for the experimental results and strongly suggest the existence of more than one binding mode.

## Figures and Tables

**Table 1:** Normalized STD NMR data from 2D-STD-TOCSYreF experiments for the fluorinated glycomimetics **1**-**3**.

Ligand	H	% STD
1	ManI-1	16
	ManI-2	42
	ManI-3	100
	ManIII-1/2	31
	ManII-1	19
	ManII-2	31
	ManII-3/6	47
	ManII-4/5	52
2	ManI-1	15
	ManI-2	33
	ManI-3	74
	ManI-4	100
	ManI-5	22
	ManIII-1	17
	ManIII-2	14
3	ManIII-1	33
	ManIII-2	31
	ManIII-3/4/5/6	47
	ManII-1	31
	ManII-2	45
	ManII-3/6	86
	ManII-4/5	100

**Table 2:** Affinities determined by competitive ^19^F-R_2_ filtered NMR experiments.

Structure	^a^ K_I_ (mM)	SD (mM)	^b^ r^2^
**1**	2.5	0.7	0.91
**1b**	1.1	0.2	0.97
**1c**	1.0	0.1	0.99
**1d**	1.1	0.2	0.98
**2**	9.6	3.5	0.77
**3**	4.0	0.6	0.98

^a^ K_I_ is determined by non-linear least squares fitting (see SI). ^b^ Coefficient of determination for the fitting.

**Table 3:** Modelled structures for MD simulations. The stability of the different complexes is also given as the association time averaged for the different MD replicas (6–12 replicas of 400 ns each). The ManI_O3-O4 pose for **1**, ManI_O3-O4 for **2** and ManII_O3-O4 for **3** are the most stable ones.

Ligand	Binding Poses	Complex Stability (ns) ^1^
**1**	ManI_O3-O4	321
	ManI_O4-O3	9
	ManII_O3-O4	27
	ManII_O4-O3	22
**2**	ManI_O3-O4	77
	ManI_O4-O3	18
**3**	ManII_O3-O4	400
	ManII_O4-O3	22
	ManIII_O3-O4	19
	ManIII_O4-O3	1

## Supplementary Materials

The following are available online at https://www.mdpi.com/1424-8247/13/8/179/s1, Figure S1: 19F-R2 filtered experiments, Figure S2: Conformational maps.; Figure S3: Selected MD frames; Figure S4: MD derived complexes association times; Figure S5: Ligand-protein interactions; Figure S6: Ligand-protein interactions for ligand 1b; Figure S7: Conformational population comparisons Table S1: Best-model STD fitting by BM-Mixer; Table S2: 1H and 19F NMR assignment of compounds 1, 2 and 3, Table S3: Best-model STD fitting by BMMixer with non-fluorinated controls.
