# Supplementary material for: The Interaction of Fluorinated Glycomimetics with DC-SIGN: Multiple Binding Modes Disentangled by the Combination of NMR Methods and MD Simulations
Source: Pharmaceuticals (Basel). 2020 Aug 4;13(8):179. doi: 10.3390/ph13080179 (PMC7463913; doi:10.3390/ph13080179)
Supplement: Supplementary file 1 [file pharmaceuticals-13-00179-s001.pdf]

Supporting information

Synthesis of **1c** and **1d**

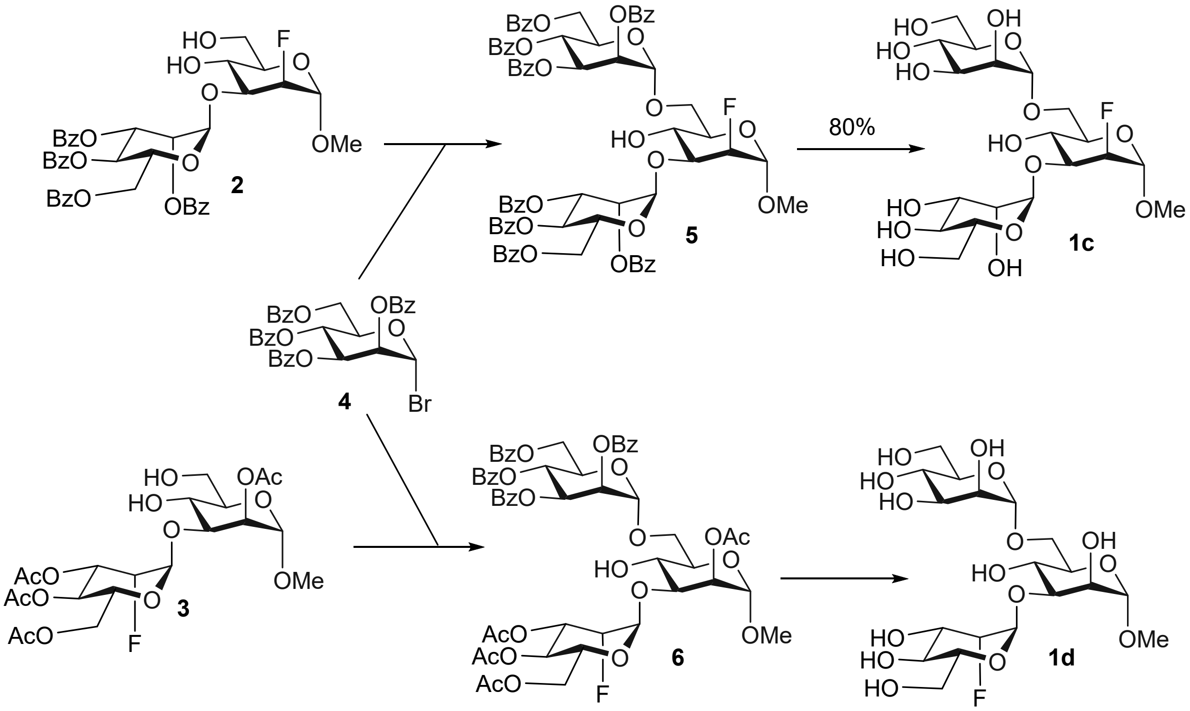

Scheme S1. Synthesis of compounds **1c** and **1d**.

Methyl 2,3,4,6-tetra-O-benzoyl-α-D-mannopyranosyl-(1→3)-[2,3,4,6-tetra-O-benzoyl-α-D-mannopyranosyl-(1→6)-]-2-deoxy-2-fluoro-α-D-mannopyranoside (5)

4,6-diol acceptor **2** and donor **4** were coupled to give trisaccharide **5** (55%). R_f 0.38 (toluene/EtOAc, 7:1). $[\alpha]_D^{20}$ -32.1 (*c* 1.0; $CHCl_3$). ^{1}H NMR (500 MHz, $CDCl_3$) δ 8.16 – 8.00 (m, 10H; H_{Ar}), 7.99 – 7.94 (m, 2H; H_{Ar}), 7.84 (m, 4H; H_{Ar}), 7.58 (m, 4H; H_{Ar}), 7.53 – 7.33 (m, 16H; H_{Ar}), 7.31 – 7.22 (m, 4H; H_{Ar}), 6.15 – 6.05 (m, 2H; H-4', H-4''), 6.01 – 5.93 (m, 2H; H-3', H-3''), 5.85 (dd, $J_{2',3'}$ = 3.3 Hz, $J_{2',1'}$ = 1.8 Hz, 1H; H-2'), 5.79 (dd, $J_{2'',3''}$ = 3.4 Hz, $J_{2'',1''}$ = 1.8 Hz, 1H; H-2''), 5.38 (d, $J_{1',2'}$ = 1.8 Hz, 1H; H-1'), 5.23 (d, $J_{1'',2''}$ = 1.8 Hz, 1H; H-1''), 5.05 – 4.89 (m, 1H; H-2), 4.81 – 4.66 (m, 4H; H-1, H-5', H-6'a, H-6''a), 4.63 (ddd, $J_{5'',4''}$ = 10.1 Hz, $J_{5'',6''a}$ = 4.5 Hz, $J_{5'',6''b}$ = 2.5 Hz, 1H; H-5''), 4.53 (m, 2H; H-6'b, H-6''b), 4.12 (m, 2H; H-4, H-6a), 4.04 – 3.85 (m, 3H; H-6b, H-3, H-5), 3.45 – 3.36 (m, 3H; OCH_3), 3.32 ppm (d, $J_{OH,4}$ = 3.5 Hz, 1H; O*H*-4). ^{13}C NMR (126 MHz, $CDCl_3$) δ 166.24, 166.05, 165.62, 165.58, 165.51, 165.44, 165.38, 165.29 (8 *C*OPh), 133.52, 133.46, 133.38, 133.33, 133.24, 133.07, 133.01, 129.96-128.25 (48 C_{Ar}), 99.95 (C-1'), 98.28 (d, *J* = 29.1 Hz; C-1), 97.63 (C-1''), 88.13 (d, *J* = 177.6 Hz; C-2), 81.42 (d, *J* = 17.1 Hz; C-3), 71.19 (C-5), 70.49 (C-2''), 70.33 (C-2'), 70.21, 70.07 (C-3'', C-3'), 69.62 (C-5'), 68.85 (C-5''), 66.86 (C-6), 67.12, 66.69 (C-4', C-4''), 65.90 (C-4), 63.04, 62.97 (C-6'', C-6'), 55.22 ppm (OCH_3). ^{19}F NMR (376 MHz, $CDCl_3$) δ -203.00 ppm (ddd, $J_{F,2}$ = 49.2 Hz, $J_{F,3}$ = 29.4 Hz, $J_{F,1}$ = 7.5 Hz; 1F, F-2). HR-MS (ESI) $[M+Na]^+$ *m/z* calcd for $C_{75}H_{65}O_{23}FNa$ 1375.3798; found 1375.3844.

Methyl α-D-mannopyranosyl-(1→3)-[α-D-mannopyranosyl-(1→6)-]-2-deoxy-2-fluoro-α-D-mannopyranoside (1c)

Compound **5** was deacylated to give **1c** (91%). R_f 0.11 (EtOAc/MeOH/water, 7:2:1). $[\alpha]_D^{20}$ + 79.6 (*c* 0.25; water). ^1H NMR (400 MHz, D_2O) δ 5.00 (as, 1H; H-1'), 4.92 – 4.74 (m, 3H; H-1, H-2, H-1''), 3.98 – 3.90 (m, 2H; H-2', 1H-6), 3.90 – 3.50 (m, 15H), 3.32 ppm (s, 3H; OCH_3). ^{13}C NMR (101 MHz, D_2O) δ 102.45 (C-1'), 99.41 (C-1''), 98.00 (d, *J* = 29.3 Hz; C-1), 88.78 (d, *J* = 172.9 Hz; C-2), 77.92 (d, *J* = 16.8 Hz; C-3), 73.27, 72.65, 70.61, 70.53, 70.30, 69.87, 69.82, 66.61 (overlapping), 65.37, 64.82, 60.88, 60.86, 55.11 ppm (OCH_3). ^{19}F NMR (376 MHz, D_2O) δ -204.15 ppm (ddd, $J_{F,2}$ = 49.1 Hz, $J_{F,3}$ = 32.9 Hz, $J_{F,1}$ = 7.3 Hz; 1F, F-2). HR-MS (ESI) $[M+Na]^+$ *m/z* calcd for $C_{19}H_{33}O_{15}FNa$ 543.1701; found 543.1687.

Methyl 3,4,6-tri-O-acetyl-2-deoxy-2-fluoro-α-D-mannopyranosyl-(1→3)-[2,3,4,6-tetra-O-benzoyl-α-D-mannopyranosyl-(1→6)-]-2-O-acetyl-α-D-mannopyranoside (6)

4,6-diol acceptor **3** and donor **4** were coupled to yield **6** (64%). R_f 0.31 (toluene/EtOAc, 2:1). $[\alpha]_D^{20}$ + 10.9 (*c* 1.0; $CHCl_3$). ^1H NMR (500 MHz, $CDCl_3$) δ 8.12 – 8.01 (m, 4H; H_{Ar}), 7.97 – 7.92 (m, 2H; H_{Ar}), 7.85 – 7.79 (m, 2H; H_{Ar}), 7.62-7.54 (m, 2H; H_{Ar}), 7.53-7.47 (m, 1H; H_{Ar}), 7.46-7.33 (m, 7H; H_{Ar}), 7.29 – 7.22 (m, 2H; H_{Ar}), 6.12 (at, $J_{4'',3''} = J_{4'',5''}$ = 10.0 Hz, 1H; H-4''), 5.94 (dd, $J_{3'',4''}$ = 10.0 Hz, $J_{3'',2''}$ = 3.2 Hz, 1H; H-3''), 5.78 (dd, $J_{2'',3''}$ = 3.2 Hz, $J_{2'',1''}$ = 1.8 Hz, 1H; H-2''), 5.40 (dd, $J_{1',F}$ = 7.3 Hz, $J_{1',2'}$ = 1.3 Hz, 1H; H-1'), 5.34 (at, $J_{4',3'} = J_{4',5'}$ = 10.1 Hz, 1H; H-4'), 5.27 (d, $J_{1''-2''}$ = 1.8 Hz, 1H; H-1''), 5.26 – 5.16 (m, 2H; H-2, H-3'), 4.88 (m, 1H; H-2'), 4.77 – 4.70 (m, 2H; H-6''a, H-1), 4.58 – 4.48 (m, 2H; H-5'', H-6''b), 4.27 (dd, $J_{6'a,6'b}$ = 12.3 Hz, $J_{6'a,5'}$ = 5.7 Hz, 1H; H-6'a), 4.20 – 4.07 (m, 5H; H-5', H-6'b, H-6a, H-4, H-3), 3.95 (dd, $J_{6b,6a}$ = 11.6 Hz, $J_{6b,5}$ = 1.9 Hz, 1H; H-6b), 3.81-3.75 (m, 1H; H-5), 3.41 (s, 3H; OCH_3), 2.83 (d, $J_{OH,4}$ = 4.6 Hz, 1H; *O*H-4), 2.16, 2.11, 2.09, 2.07 ppm (4s, 12 H; 4 $OCOCH_3$). ^{13}C NMR (125 MHz, $CDCl_3$) δ 170.70, 170.56, 170.14, 169.56, (4 $OCOCH_3$), 166.30, 165.49, 165.46, 165.20 (4 *C*OPh), 133.43, 133.41, 133.18, 133.08, 129.82 -128.29 (24 C_{Ar}), 98.86 (*J* = 29.9 Hz; C-1'), 98.51 (C-1), 98.07 (C-1''), 86.79 (*J* = 180.0 Hz; C-2'), 77.13, 71.65 (C-5), 71.25 (C-2), 70.30 (C-2''), 70.00 (C-3''), 69.85 (*J* = 16.8 Hz; C-3'), 69.43, 68.88 (C-5''), 67.12 (C-4''), 66.96, 66.39 (C-6), 65.56 (C-4'), 62.95 (C-6''), 62.18 (C-6'), 55.13 (OCH_3), 20.75, 20.73, 20.67, 20.59 ppm (4 $OCOCH_3$). ^{19}F NMR (376 MHz, $CDCl_3$) δ -203.61 ppm (ddd, $J_{F-2'}$ = 49.4 Hz, $J_{F-3'}$ = 28.2 Hz, $J_{F-1'}$ = 7.3 Hz; 1F, 2'-F). HR-MS (ESI) $[M+Na]^+$ *m/z* calcd for $C_{55}H_{57}O_{23}F$ 1127.3172; found 1127.3116.

Methyl 2-deoxy-2-fluoro-α-D-mannopyranosyl-(1→3)-[α-D-mannopyranosyl-(1→6)-]-α-D-mannopyranoside (1d)

Compound **6** was deacylated to give trisaccharide **1d** (80%). R_f 0.15 (EtOAc/MeOH/H_2O, 7:2:1). $\alpha]_D^{20}$ + 99.0 (*c* 0.5; water). ^1H NMR (500 MHz, D_2O) δ 5.16 (dd, $J_{1',F}$ = 8.0 Hz, $J_{1',2'}$ = 1.8 Hz, 1H; H-1'), 4.80 – 4.64 (m, 2H; H-1'', H-2'), 4.57 (as, 1H; H-1), 3.94 (as, 1H; H-2) 3.89 – 3.71 (m, 7H), 3.70 – 3.47 (m, 9H), 3.25 ppm (s, 1H; OCH_3). ^{13}C NMR (126 MHz, D_2O) δ 100.96 (C-1), 99.44 (C-1''), 99.32 (d, *J* = 30.4 Hz; C-1'), 89.55 (d, *J* = 172.3 Hz; C-2'), 79.06, 73.27, 72.71, 70.81, 70.60, 69.96, 69.60 (d, *J* = 17.5 Hz; C-3'), 69.49, 66.73, 66.66, 65.52, 65.21, 60.95, 60.50, 54.85 ppm (OCH_3). ^{19}F NMR (376 MHz, D_2O) -204.73 ppm (ddd, $J_{F,2'}$ = 49.3 Hz, $J_{F,3'}$ = 31.6 Hz, $J_{F,1'}$ = 8.0 Hz; 1F; F-2'). HR-MS (ESI) $[M+Na]^+$ *m/z* calcd for $C_{19}H_{33}O_{15}FNa$ 543.1701; found 543.1709.

NMR experiments

Table S2: 1H and 19F NMR assignment of compounds **1**, **2** and **3**

Table S2a. 2-F-Man_3, compound **1.**

Position	***ManI***		***ManII***		***ManIII***	
	^{1}H	^{19}F	^{1}H	^{19}F	^{1}H	^{19}F
1	5.27		5.06		4.90	
2	4.81	-204.86	4.75	-205.97	4.89	-204.21
3	3.87		3.83		3.92	
4	3.66		3.66		3.86	
5	3.75		3.66		3.82	
6, 6'	3.82, 3.73		3.82, 3.73		4.03, 3.72	
Me					3.37	

Table S2b. 2-F-$Man_{2,\alpha1\text{-}3}$, compound **2.**

Position	***ManI***		***ManIII***	
	^{1}H	^{19}F	^{1}H	^{19}F
1	5.28		4.91	
2	4.81	-204.82	4.87	-204.03
3	3.87		3.92	
4	3.65		3.75	
5	3.75		3.65	
6, 6'	3.81, 3.73		3.83, 3.73	
Me			3.37	

Table S2c. 2-F-$Man_{2,\alpha1\text{-}6}$, compound **3.**

Position	***ManI***		***ManIII***	
	^{1}H	^{19}F	^{1}H	^{19}F
1	5.07		4.90	
2	4.75	-205.86	4.70	-206.00
3	3.83		3.76	
4	3.66		3.73	
5	3.66		3.75	
6, 6'	3.83, 3.73		4.00, 3.74	
Me			3.37	

^{19}F-R_2 filtered experiments

The K_D of compounds **1**, **1b-d**, **2** and **3** was estimated applying a ^{19}F-R_2 filtered approach. 6-F-ManαOMe, which weakly binds to DC-SIGN, was selected as the spy molecule. Relaxation rates R_2 were determined employing a CPMG pulse sequence, by fitting the observed ^{19}F signal intensity to the exponential decay curve:

$$I(t) = I_0 e^{-tR_2} = I_0 e^{-n2\tau R_2} \tag{1}$$

where $I(t)$ refers to intensity at time t, I_0 is intensity at $t = 0$, and R_2 is the transversal relaxation rate ($R_2 = 1/T_2$).

In the limit of fast-exchange where the exchange contribution to the observed transversal relaxation rate $R_{2,obs}$ is insignificant (Figure S1, a)), the following equations apply:

$$R_{2,obs} = R_{2,f} + \left(R_{2,b} - R_{2,f}\right) p_b \tag{2}$$

$$p_b = \frac{[P]_T + [L]_T + K_D - \sqrt{([P]_T + [L]_T + K_D)^2 - 4[P]_T[L]_T}}{2[L]_T} \tag{3}$$

where $[P]_T$ and $[L]_T$ are the total protein and ligand concentrarion respectively, $[R]_{2,f}$ and $[R]_{2,b}$ are the relaxation rates in the free and bound states, p_b is the fraction of bound ligand and K_D the dissociation constant of the protein-ligand complex. $[R]_{2,f}$ of 6-F-ManαOMe was measured in absence of the lectin, and Equation 2 was used to estimate $K_D$and $[R]_{2,b}$ for the complex (Figure S1, b)).

Then, K_I of compounds **1**, **1b-d**, **2** and **3** was measured in a competitive manner. $R_{2,obs}$ of 6-F-ManαOMe (spy molecule) in solution with DC-SIGN ECD was monitored at 5 different competitor concentrations ($[I]$) in each case with a fixed $[P]_T/[L]_T$ ratio (Figure S1, c)), to derive K_I by fitting to Equation 2 with p_b as defined in Equations (4) and (5) (Table 2):

$$p_b = \frac{2\cos(\theta/3)\sqrt{a^2 - 3b} - a}{3K_D + 2\cos(\theta/3)\sqrt{a^2 - 3b} - a} \tag{4}$$

$$\theta = \cos^{-1}\left(\frac{-2a^3 + 9ab - 27c}{2\sqrt{(a^2 - 3b)^3}}\right),\ a = K_D + K_I + [L]_T + [I]_T - [P]_T,$$
$$b = ([I]_T - [P]_T)K_D + ([L]_T - [P]_T)K_D + K_I K_D,\ c = -K_I K_D [P]_T \tag{5}$$

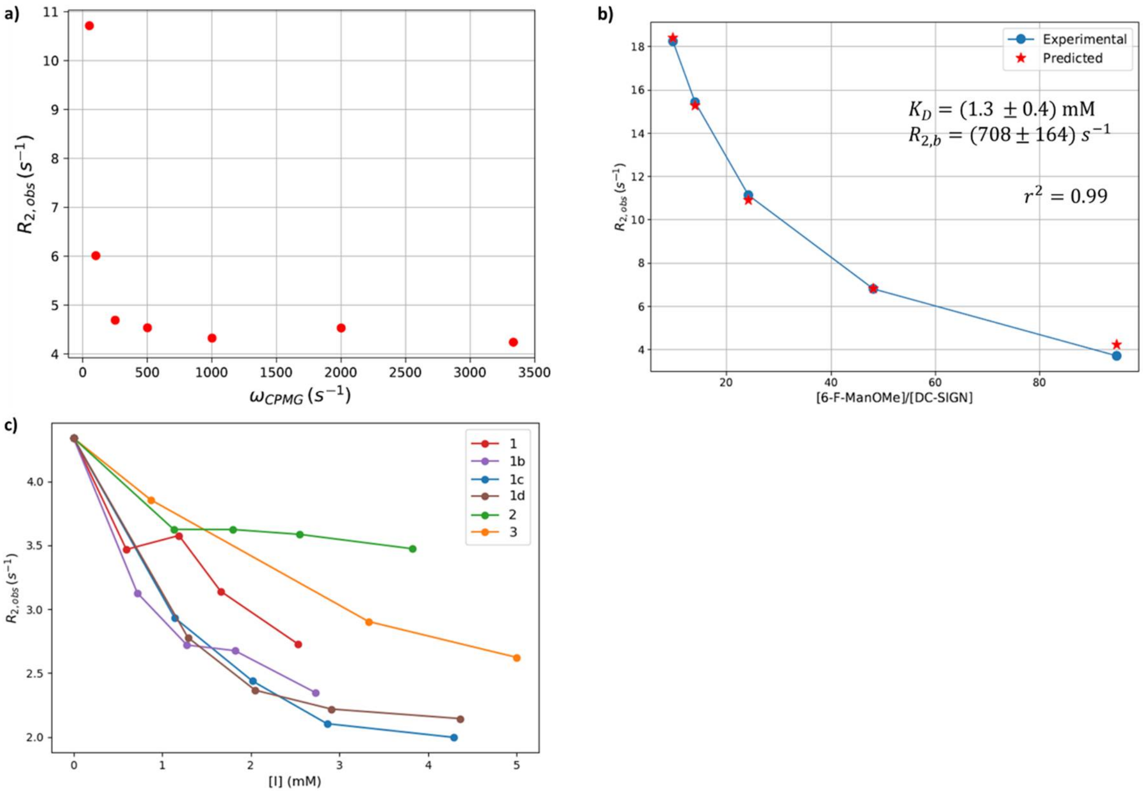

Figure S1. **^{19}F-R$_2$ filtered experiments**. a) Relaxation dispersion experiment for 6-F-ManαOMe (the spy molecule). $R_{2,obs}$ of the ^{19}F nucleus is measured for different values of ω_{CPMG}. Ligand and protein sample concentrations were: [6-F-ManαOMe] = 400 µM, [DC-SIGN (CRDs)] = 10 µM (counting concentration of CRDs, i.e, 4 CRDs per DC-SIGN ECD tetramer). For $\omega_{CPMG} > 1000\ s^{-1}$, there is virtually negligible exchange contribution to R_2. Therefore, all the subsequent R_2 filtered experiments were carried out with $\tau_{CPMG} = 1/\omega_{CPMG}$ = 1 ms. b) K_D determination of 6-F-ManαOMe with DC-SIGN. $R_{2,obs}$ was measured for increasing amounts of [6-F-ManαOMe]/[DC-SIGN (CRDs)] (blue dots). K_D and $R_{2,b}$ were obtained from fitting to Equation 2, which is valid in the fast-exchange regime (R_{ex} = 0) [39a]. The predicted values at each [6-F-ManαOMe]/[DC-SIGN (CRDs)] are shown as red stars for comparison c) Titration curves showing the variation in ^{19}F-$R_{2,obs}$ of the spy molecule 6-F-ManαOMe, when increasing amounts of the competitors (I) **1**, **1b-d**, **2** and **3** are added to a mixture [6-F-ManαOMe] = 400 µM, [DC-SIGN (CRDs)] = 10 µM.

MD simulations

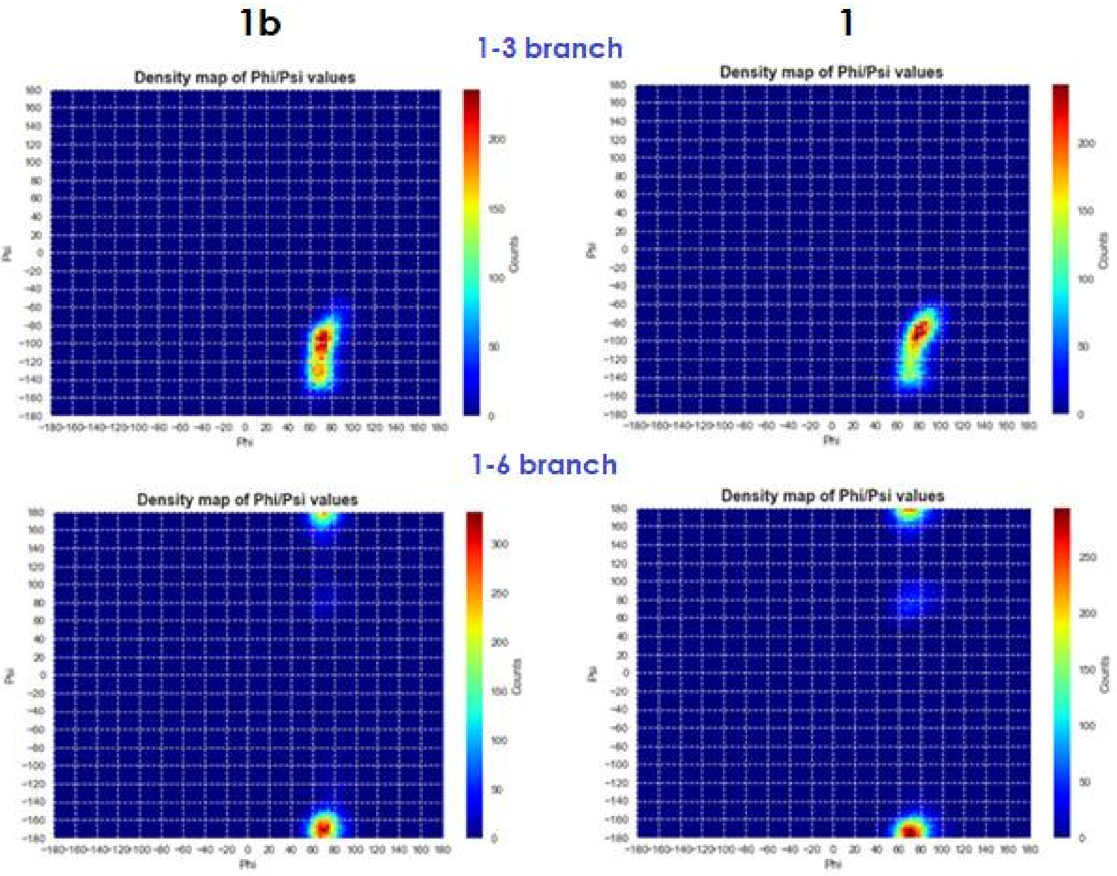

Figure S2. **Conformational maps.** Density of conformers populations around ϕ/ψ torsion angles computed for **1** and **1b** during a 500 ns MD simulation in explicit TIP3P water. ϕ and ψ torsion angles are defined as O5(*i*)-C1(*i*)-O*n*(*i*-1)-C*n*(*i*-1) and C1(*i*)-O*n*(*i*-1)-C*n*(*i*-1)-C(*n*-1)(*i*-1) respectively, where *n* indicates ring position and *i* a given residue. For **1b**, the GLYCAM 06-j [40] forcefield was employed, whereas GAFF2 [44] was used for **1**. The MD protocol in both simulations is described in the Materials and Methods section. The maps are fairly similar, independently of the employed force field.

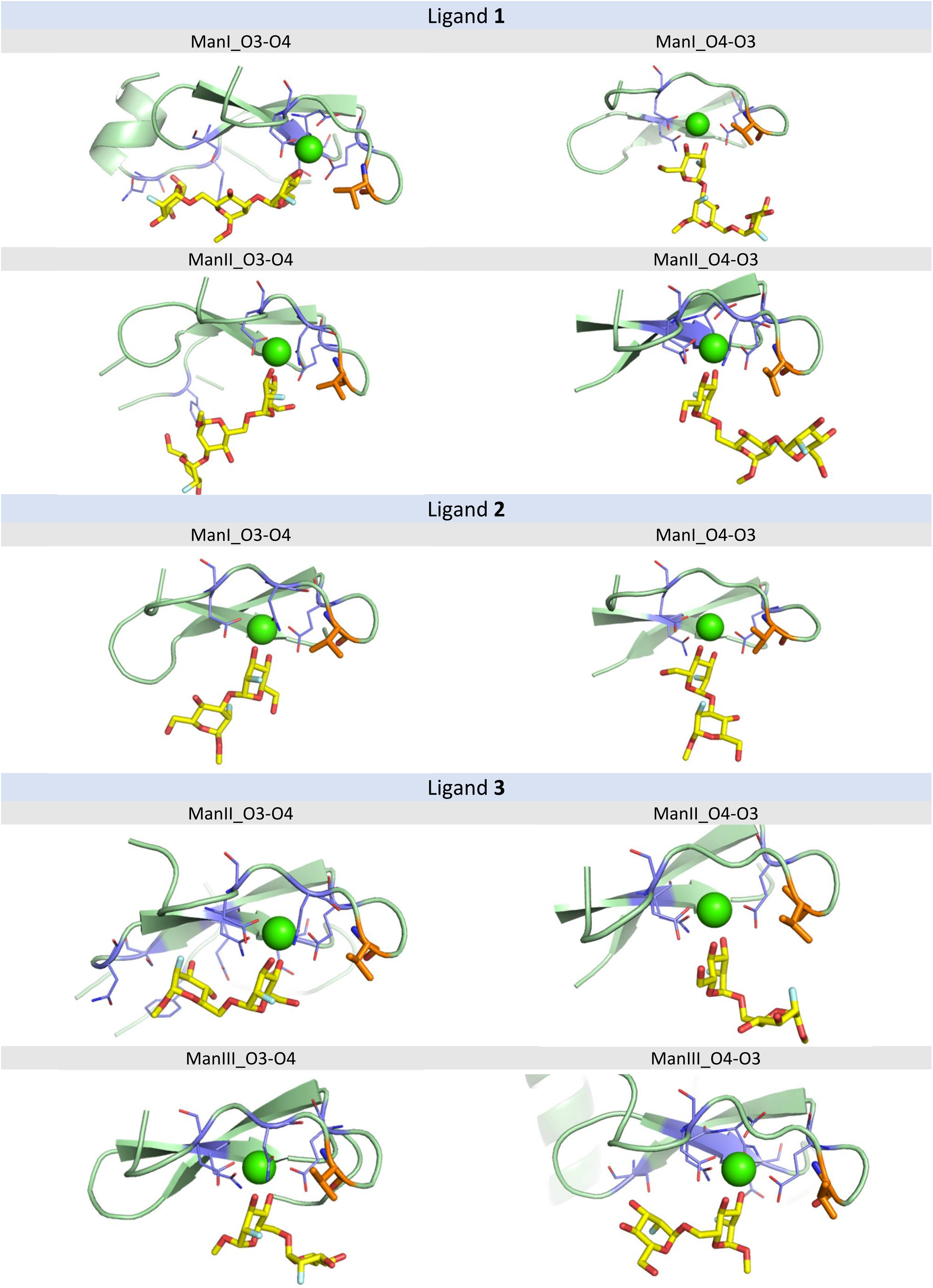

Figure S3. **Selected MD frames:** Representative optimized structures of each proposed binding mode for **1**, **2** and **3** in complex with DC-SIGN after system minimization of the first MD replica.

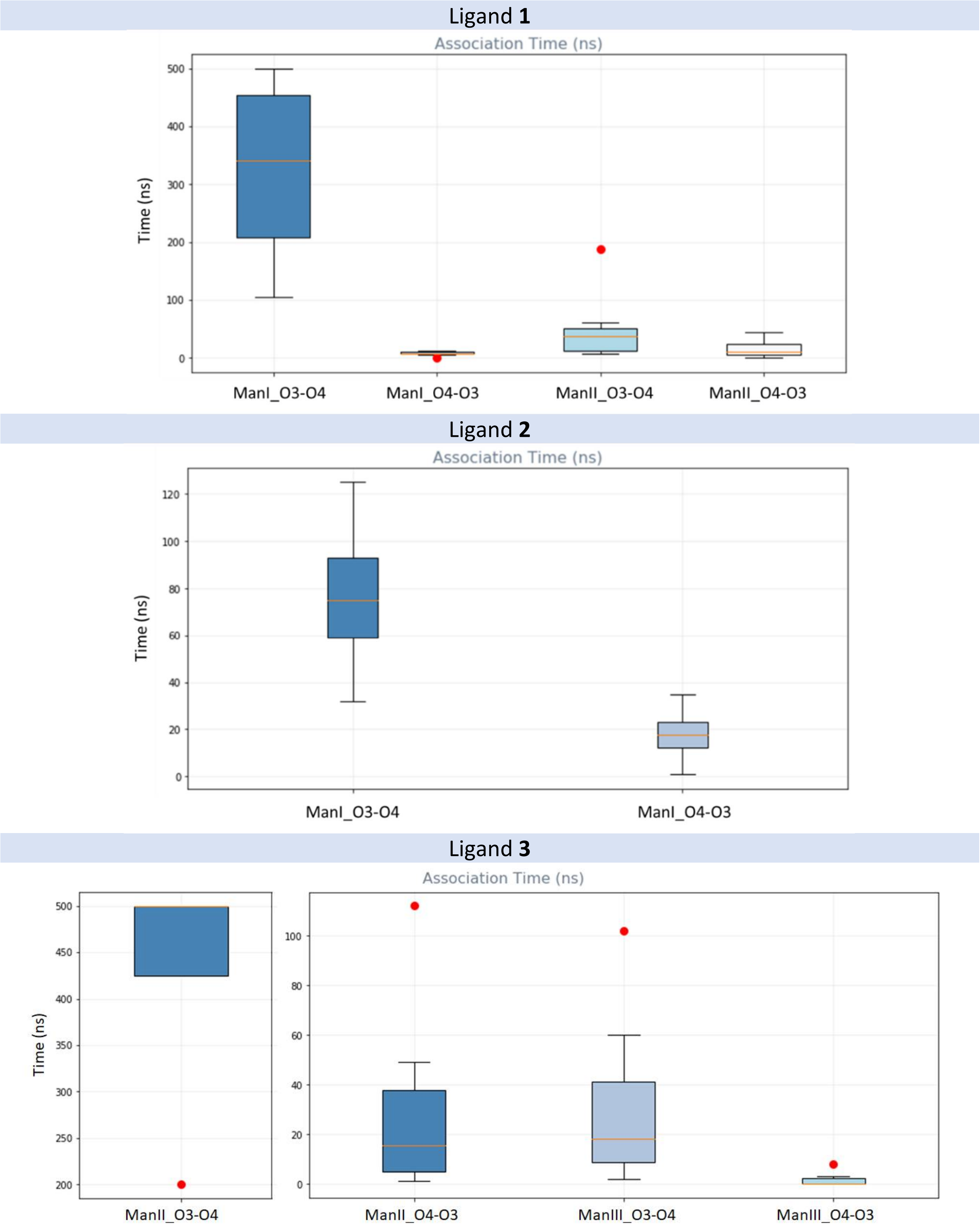

Figure S4. MD derived complexes association times: Box plot representation of association times observed in the MD simulations of the different ligand-protein complexes. The number of MD replicas ran in each case varies from 6 to 12, depending on the variability observed. Outliers are represented as red dots.

Ligand **1**

ManI_O3-O4

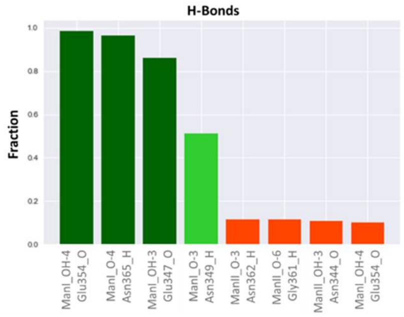

Other Interactions			
**Type**	**Ligand Group**	**Amino acid**	**Fraction (%)**
CH-Pi	ManIII_H6/H6’	Phe313	8

ManI_O4-O3

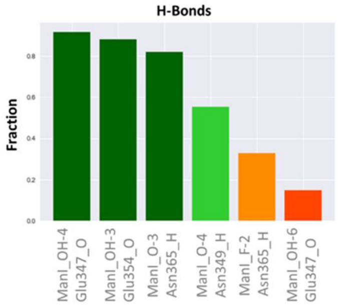

Other Interactions			
**Type**	**Ligand Group**	**Amino acid**	**Fraction (%)**
VdW	ManI_H-3	Val351	21
VdW	ManII_H-4	Val351	29
VdW	ManIII_H-3	Val351	27
VdW	ManIII_H-5	Val351	23

ManII_O3-O4

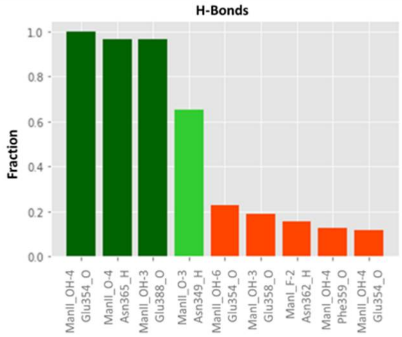

Other Interactions			
**Type**	**Ligand Group**	**Amino acid**	**Fraction (%)**
VdW	ManIII_H-4	Val351	20

ManII_O4-O3

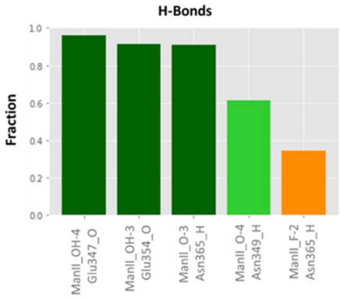

Other Interactions			
**Type**	**Ligand Group**	**Amino acid**	**Fraction (%)**
VdW	ManI_H-3	Val351	50
VdW	ManI_H-5	Val351	15
VdW	ManII_H-3	Val351	40
VdW	ManIII_H-3	Val351	10
VdW	ManIII_H-4	Val351	13

Ligand **2**

ManI_O3-O4

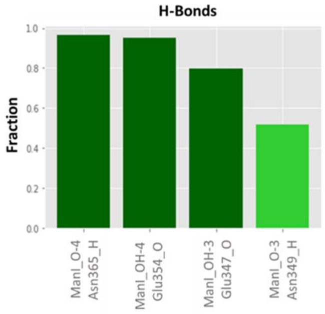

ManI_O4-O3

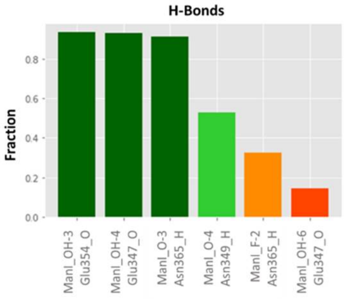

Other Interactions			
**Type**	**Ligand Group**	**Amino acid**	**Fraction (%)**
VdW	ManI_H-3	Val351	12
VdW	ManII_H-4	Val351	14

Ligand **3**

ManII_O3-O4

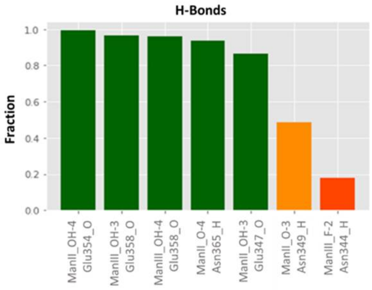

Other Interactions			
**Type**	**Ligand Group**	**Amino acid**	**Fraction (%)**
CH-Pi	ManIII_H-3	Phe313	70

ManII_O4-O3

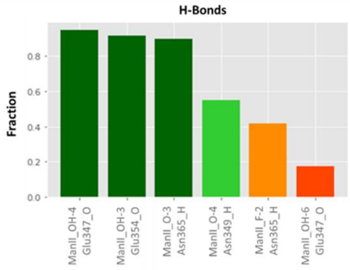

Other Interactions			
**Type**	**Ligand Group**	**Amino acid**	**Fraction (%)**
VdW	ManII_H-3	Val351	27

ManIII_O3-O4

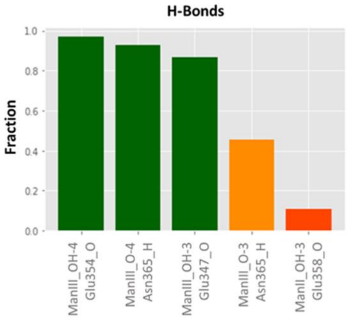

ManIII_O4-O3

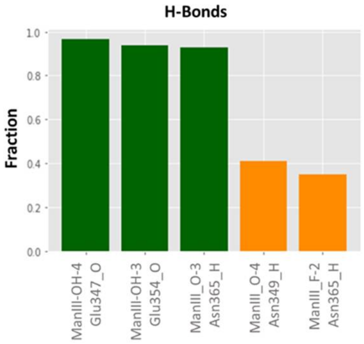

Figure S5. **Ligand-protein interactions**: Significant ligand-protein interactions computed during the MD replicas. The fraction axis shows the percentage of the simulation time that the interaction is found.

Hydrogen-bonds are accounted from the MD trajectories based on distance and angle criteria: d_(A-H-D) < 3 Å and (A-H-D)̂ <130°, where H refers to the coordinates of the hydrogen atom, D and A the hydrogen bond donor and acceptor respectively. Similarly, CH-Pi interactions are accounted by the distance of the aromatic ring-center to the hydrogen atom involved in the interaction according to d_(Ring-H) < 3 Å, as well as the C/H/Ring-center angle (C-H-Ring)̂ >120°. Van der Waals interactions are considered when the interatomic distance of the atoms involved is lower than 1.2 times the sum of the VdW radii of the atoms.

Ligand **1b**

ManI_O3-O4

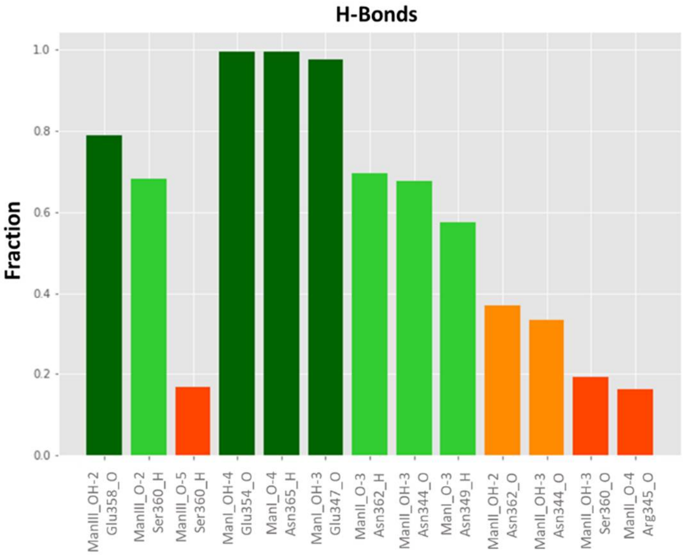

Other Interactions			
**Type**	**Ligand Group**	**Amino acid**	**Fraction (%)**
CH-Pi	ManIII_H1	Phe313	31

Figure S6. **Ligand-protein interactions for ligand 1b**: Significant ligand-protein interactions computed during the MD of DC-SIGN bound to **1b** *via* ManI_O3-O4. All interactions are accounted in the same way as described in Figure S5

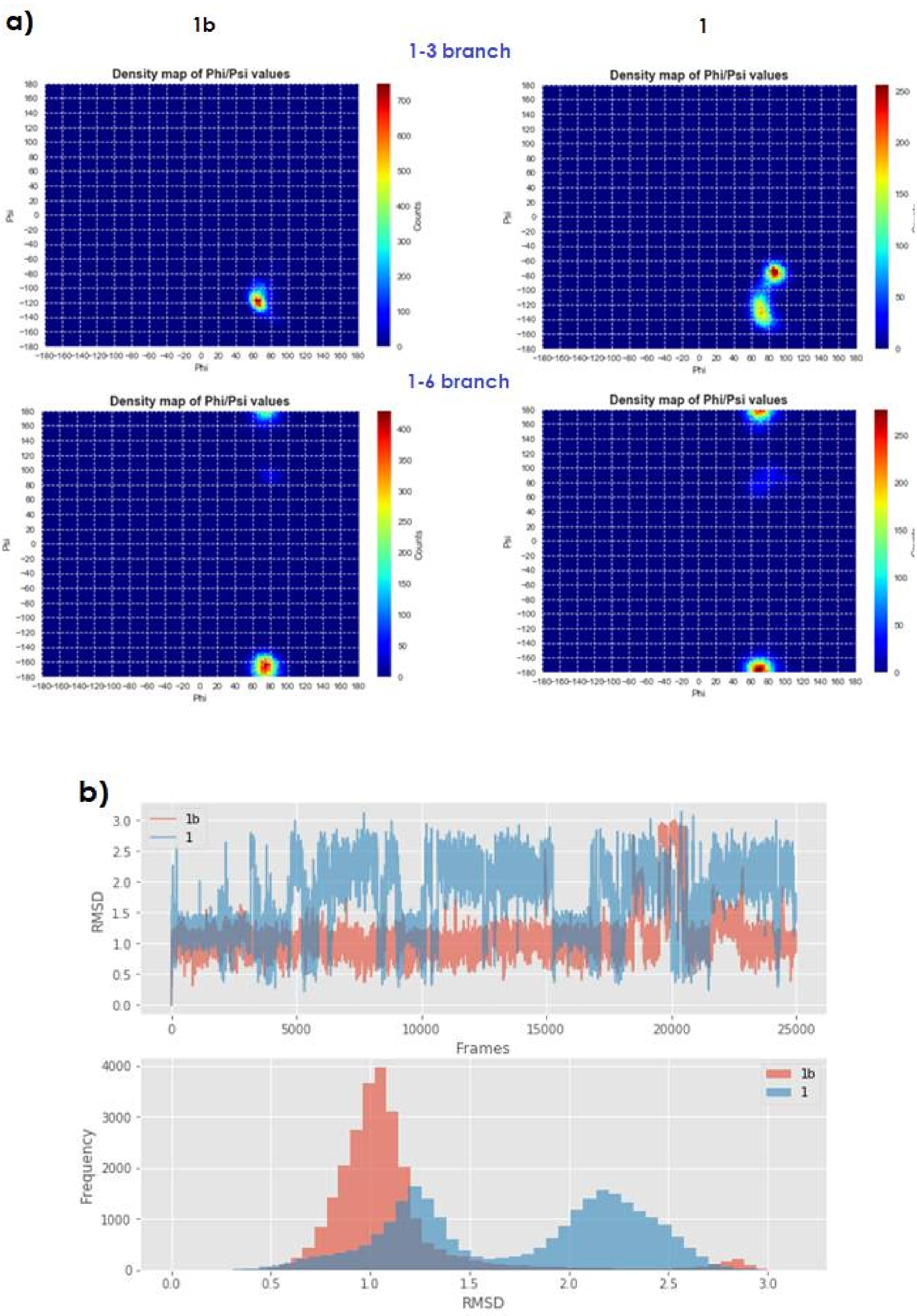

Figure S7. Conformational population comparisons: Comparison the populations around ϕ/ψ torsion angles (*a)*) and the RMSD (*b)*) of the ligands computed in a 500 ns MD simulation of **1** and **1b** bound *via* ManI_O3-O4 to DC-SIGN. It can be observed the larger mobility of the glycomimetic **1** with respect to the natural trimannose **1b** at the binding site when bound through the same pose.

CORCEMA-ST

The CORCEMA-ST script was ran sequentially for 400-800 frames extracted from each MD simulation trajectory. The same experimental parameters employed in the STD-NMR experiments were used in the calculations: [DC-SIGN] = 9.14 uM, [Ligand] = 1.4 mM, 2 s saturation time. Different k_{on} values in the range of 10^5-10^8 M^{-1} $s^{-1}$and K_D 0.5-3 mM were tested with all the complexes, giving rise to very similar normalized calculated STD profiles. Thus, a k_{on} of 10^6 M^{-1} s^{-1}, of the same order of other sugar-lectin systems [1,2], was used for all the models. K_D was set to 1 mM, similar to the observed K_D of other Man derivatives in complex with DC-SIGN [3-5]. An instantaneous irradiation of the aliphatic receptor residues Ile, Leu, and Val methylgroups to account for the selective on-resonance irradiation of the STD-NMR experiment, 0.85 ppm, was used. The size of the relaxation matrix was adjusted using a distance cutoff, *d*, of 10 Å away from any ligand atom, since virtually the same STD profiles were obtained for larger values, while some differences appeared when d < 10 Å. The value of the order parameter S^2 and the methyl group internal correlation time τ_m were set to 0.85 and 10 ps respectively, as previously described [6]. A typical value for the free ligand correlation time τ_L = 0.5 ns was used, whereas for the bound ligand a correlation time assuming a tetrameric protein of globular shape was estimated as τ_b = 85 ns.

Since CORCEMA-ST does not recognize ^{19}F as an active nucleus in the relaxation matrix, the effect of the presence of an active nuclei at position C-2 in the 2-F compounds **1**, **2** and **3**, was assessed by substituting all fluorine atoms by hydrogens in each analogue, while keeping the original C-F distance. Then, CORCEMA-ST calculations were ran with the same parameters described in the previous paragraph. This way, the ^{1}H nucleus is used as a probe to simulate the most pronounced expectable effect (since it can give rise to homonuclear cross-relaxation) on the observed STD signals. Remarkably, it was found that the predicted best fitting models with *BM-Mixer* are in general unaffected by the presence of the active nucleus at C-2 for the three ligands (see Table S3).

Best-model STD fitting

BM-Mixer is able to find the best combination of frames (in %) from different MD trajectories explaining the experimental STD-NMR data. For the program to work properly, it is important to use a list of experimental STDs only containing reliable assigned peaks. In this work, we used the list provided in Table 1 in the main text, with the exception of compound **3**. For compound **3**, the experimental STD cross peak observed for Man III at 3.7 ppm could correspond to H-3, H-4, H-5 or H-6'. As accounting the measured STD intensity as the sum of the individual contribution from four H atom would potentially introduce noise in the search (see *CORCEMA-ST and best-model STD fitting* heading in the experimental section), the corresponding STD peak was not taken into account in the search for best-model fitting showed in Figure 4 and Table S2.

There are two main parameters that must be set in a *BM-Mixer* run: *mix_leap* and *search_iterator*. *mix_leap* defines the minimum percentage of frames to be used from each trajectory to explore the different combinations. For example, setting *mix_leap* = 5 allows the program to combine frames from different trajectories using a minimum of 5 % of the frames. Although it depends on the number of frames and binding modes (trajectories) to work with, typically a value of *mix_leap* = 10 is sufficient to get accurate enough results (according to NOE R-$Factor_{Rel}$) in a decent amount of time. *search_iterator* specify how many times the program is run before computing the final NOE R-$Factor_{Rel}$ averages. Every time a new iteration start (when

search_iterator > 1), the frames used in each combination are randomly selected, so that the larger the value of *search_iterator*, the better sampling of the trajectory-space is done. In general, we have found that for the studied systems, when using 400-800 frames of each binding mode trajectory, best NOE R-Factor$_{Rel}$ averages are similar when setting *search_iterator* > 15.

Table S1. Best-model STD fitting by *BM-Mixer*: Top 3 best-model STD fitting results for each ligand, found by *BM-Mixer*. For ligands **1** and **3**, 800 frames from each simulated binding mode were used in the calculations, while 400 frames were employed for **2**. *mix_leap* was set to 10 for ligands **1** and **3**, and to 5 for **2**; a *search_iterator* of 30 was used in all cases.

Ligand **1**				
ManI_O3-O4 (%)	ManI_O4-O3 (%)	ManII_O3-O4 (%)	ManII_O4-O3 (%)	NOE R-Factor$_{Rel}$
60	0	0	40	0.2010
50	0	0	50	0.2201
40	0	10	50	0.2238

Ligand **2**		
ManI_O3-O4 (%)	ManI_O4-O3 (%)	NOE R-Factor$_{Rel}$
65	35	0.1380
60	40	0.1396
70	30	0.1575

Ligand **3**			
ManII_O3-O4 (%)	ManII_O4-O3 (%)	ManIII_O3-O4 (%)	NOE R-Factor$_{Rel}$
50	10	40	0.1470
50	0	50	0.1482
40	10	50	0.1496

Table S3. Best-model STD fitting by *BM-Mixer* with non-fluorinated control Top 3 best-model STD fitting results found by *BM-Mixer* for each ligand-control CORCEMA-ST calculated STD. Ligand-controls were built by substituting all fluorine atoms in the MD trajectories by hydrogens, and then computing CORCEMA-ST on those. The same *BM-Mixer* set up described in Table S2 was used.

Ligand **1**				
ManI_O3-O4 (%)	ManI_O4-O3 (%)	ManII_O3-O4 (%)	ManII_O4-O3 (%)	NOE R-Factor$_{Rel}$
60	0	0	40	0.2143
40	0	10	50	0.2212
40	0	10	50	0.2368

Ligand **2**		
ManI_O3-O4 (%)	ManI_O4-O3 (%)	NOE R-Factor$_{Rel}$
70	30	0.1467
65	35	0.1538
75	25	0.1681

Ligand **3**			
ManII_O3-O4 (%)	ManII_O4-O3 (%)	ManIII_O3-O4 (%)	NOE R-Factor$_{Rel}$
50	0	50	0.1555
40	10	50	0.1645
50	10	40	0.1654

References

1. Scharenberg, M.; Jiang, X.; Pang, L.; Navarra, G.; Rabbani, S.; Binder, F.; Schwardt, O.; Ernst, B. Kinetic properties of carbohydrate-lectin interactions: FimH antagonists. *ChemMedChem* **2014**, *9*, 78–83.
2. Milton, J.D.; Fernig, D.G.; Rhodes, J.M. Use of a biosensor to determine the binding kinetics of five lectins for Galactosyl-N-acetylgalactosamine. *Glycoconj. J.* **2001**, *18*, 565–569.
3. Holla, A.; Skerra, A. Comparative analysis reveals selective recognition of glycans by the dendritic cell receptors DC-SIGN and Langerin. *Protein Eng. Des. Sel.* **2011**, *24*, 659–669.
4. Bordoni, V.; Porkolab, V.; Sattin, S.; Thépaut, M.; Frau, I.; Favero, L.; Crotti, P.; Bernardi, A.; Fieschi, F.; Di Bussolo, V. Stereoselective innovative synthesis and biological evaluation of new real carba analogues of minimal epitope Manα(1,2)Man as DC-SIGN inhibitors. *RSC Adv.* **2016**, *6*, 89578–89584.
5. Reina, J.J.; Sattin, S.; Invernizzi, D.; Mari, S.; Martinez-Prats, L.; Tabarani, G.; Fieschi, F.; Delgado, R.; Nieto, P.M.; Rojo, J.; et al. 1,2-mannobioside mimic: Synthesis, DC-SIGN interaction by NMR and docking, and antiviral activity. *ChemMedChem* **2007**, *2*, 1030–1036.
6. Jayalakshmi, V.; Krishna. N.R. Complete relaxation and conformational exchange matrix (CORCEMA) analysis of intermolecular saturation transfer effects in reversibly forming ligand-receptor complexes. *J. Magn. Reson.* **2002**, *155*, 106–118.
